# mTOR inhibition overcomes RSK3-mediated resistance to BET inhibitors in small cell lung cancer

**DOI:** 10.1172/jci.insight.156657

**Published:** 2023-03-08

**Authors:** Anju Kumari, Lisa Gesumaria, Yan-Jin Liu, V. Keith Hughitt, Xiaohu Zhang, Michele Ceribelli, Kelli M. Wilson, Carleen Klumpp-Thomas, Lu Chen, Crystal McKnight, Zina Itkin, Craig J. Thomas, Beverly A. Mock, David S. Schrump, Haobin Chen

**Affiliations:** 1 Thoracic Surgery Branch and; 2 Laboratory of Cancer Biology and Genetics, Center for Cancer Research, National Cancer Institute, NIH, Bethesda, Maryland, USA.; 3 Division of Preclinical Innovation, National Center for Advancing Translational Sciences, NIH, Rockville, Maryland, USA.; 4 Lymphoid Malignancies Branch, Center for Cancer Research, National Cancer Institute, NIH, Bethesda, Maryland, USA.

**Keywords:** Oncology, Drug screens, Epigenetics

## Abstract

Small cell lung cancer (SCLC) is a recalcitrant malignancy with limited treatment options. Bromodomain and extraterminal domain inhibitors (BETis) have shown promising preclinical activity in SCLC, but the broad sensitivity spectrum limits their clinical prospects. Here, we performed unbiased high-throughput drug combination screens to identify therapeutics that could augment the antitumor activities of BETis in SCLC. We found that multiple drugs targeting the PI-3K–AKT–mTOR pathway synergize with BETis, among which mTOR inhibitors (mTORis) show the highest synergy. Using various molecular subtypes of the xenograft models derived from patients with SCLC, we confirmed that mTOR inhibition potentiates the antitumor activities of BETis in vivo without substantially increasing toxicity. Furthermore, BETis induce apoptosis in both in vitro and in vivo SCLC models, and this antitumor effect is further amplified by combining mTOR inhibition. Mechanistically, BETis induce apoptosis in SCLC by activating the intrinsic apoptotic pathway. However, BET inhibition leads to RSK3 upregulation, which promotes survival by activating the TSC2-mTOR-p70S6K1-BAD cascade. mTORis block this protective signaling and augment the apoptosis induced by BET inhibition. Our findings reveal a critical role of RSK3 induction in tumor survival upon BET inhibition and warrant further evaluation of the combination of mTORis and BETis in patients with SCLC.

## Graphical abstract

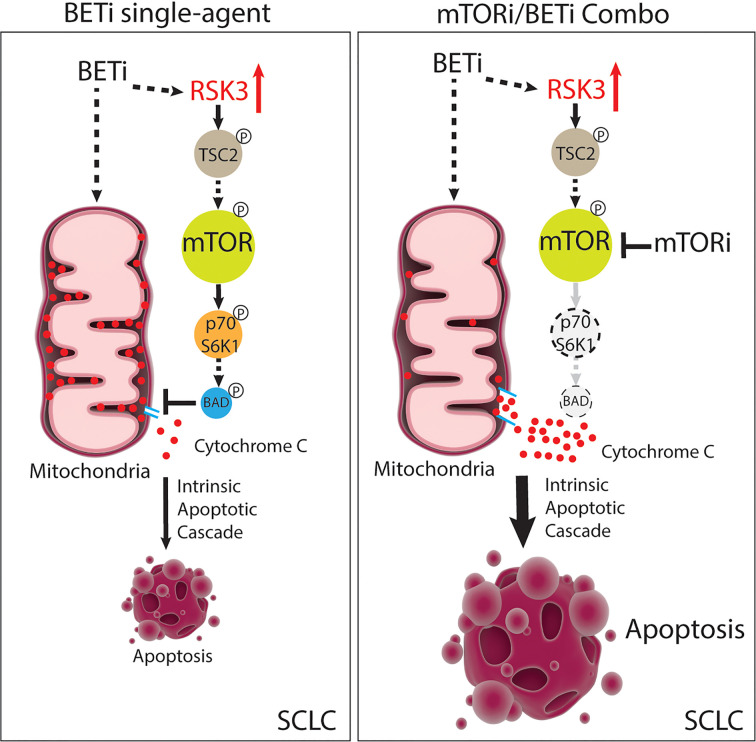

## Introduction

Small cell lung cancer (SCLC) is a recalcitrant malignancy with limited treatment options (1). There was no improvement in its 2-year survival between 2001 and 2014 due to a lack of treatment advances for nearly 2 decades (2). Several new therapeutics, including immune checkpoint inhibitors and lurbinectedin, were recently approved for this malignancy, but only a subset of patients would benefit (3). Therefore, there is an unmet need to develop novel therapies for SCLC.

A potential therapeutic candidate for SCLC is bromodomain and extra-terminal domain inhibitors (BETis), which target the BET family proteins, namely BRD2, BRD3, BRD4, and BRDT. The primary function of the BET family proteins is gene transcription regulation. BETis bind to the bromodomains of BET family proteins and dissociate them from active chromatin, causing suppression of gene transcription. Because BETis only decrease the expression of a subset of genes, particularly those related to cell lineage and driver oncogenes (4), there has been considerable interest in applying this class of drugs for cancer treatment.

Previous studies reported that mouse SCLC was exquisitely susceptible to BETis (5), but human SCLC lines had a much broader range of sensitivity (6). We recently found that a subset of SCLC expressing NEUROD1 (the SCLC-N subtype) is particularly susceptible to BETis due to the dependence of NEUROD1 transactivation on BET family proteins (7). However, BETi has only modest antitumor activity in the SCLC-N subtype tumors in vivo (7), which necessitates a combinatorial strategy to augment its antitumor effects in both SCLC-N and other molecular subtypes of SCLC. Several previous studies have reported that inhibitors targeting PARP, HDAC6, or BCL2 synergized with BETis in SCLC (8–11). However, an unbiased screen of the existing oncological therapeutics in combination with BETis has not been reported in SCLC, and the mechanisms underlying BETi resistance remain largely unknown.

The PI-3K–AKT–mTOR pathway is commonly activated in cancer, and a recent shRNA library screen identified mTOR as an essential kinase in a subset of SCLC (12). However, a phase II trial found everolimus, an mTOR inhibitor (mTORi) approved for multiple neoplasms, had only limited antitumor activity in relapsed SCLC, suggesting a need to combine this drug with other therapeutics (13). Interestingly, in breast and prostate cancer cell lines, inhibition of mTOR complex 1 (mTORC1) causes a feedback activation of RTKs, which turns on AKT signaling and promotes survival (14, 15). BETi increases the mTORi antitumor activities in these cell lines by preventing RTK upregulation and blocking this feedback loop (16, 17). However, to our knowledge, the combination of mTORis and BETis has not been explored in SCLC.

In this study, we performed high-throughput drug combination screens to identify potential therapeutics synergizing with BETis in SCLC in an unbiased manner.

## Results

### mTORis synergize with BETis in vitro.

**Figure 1:**
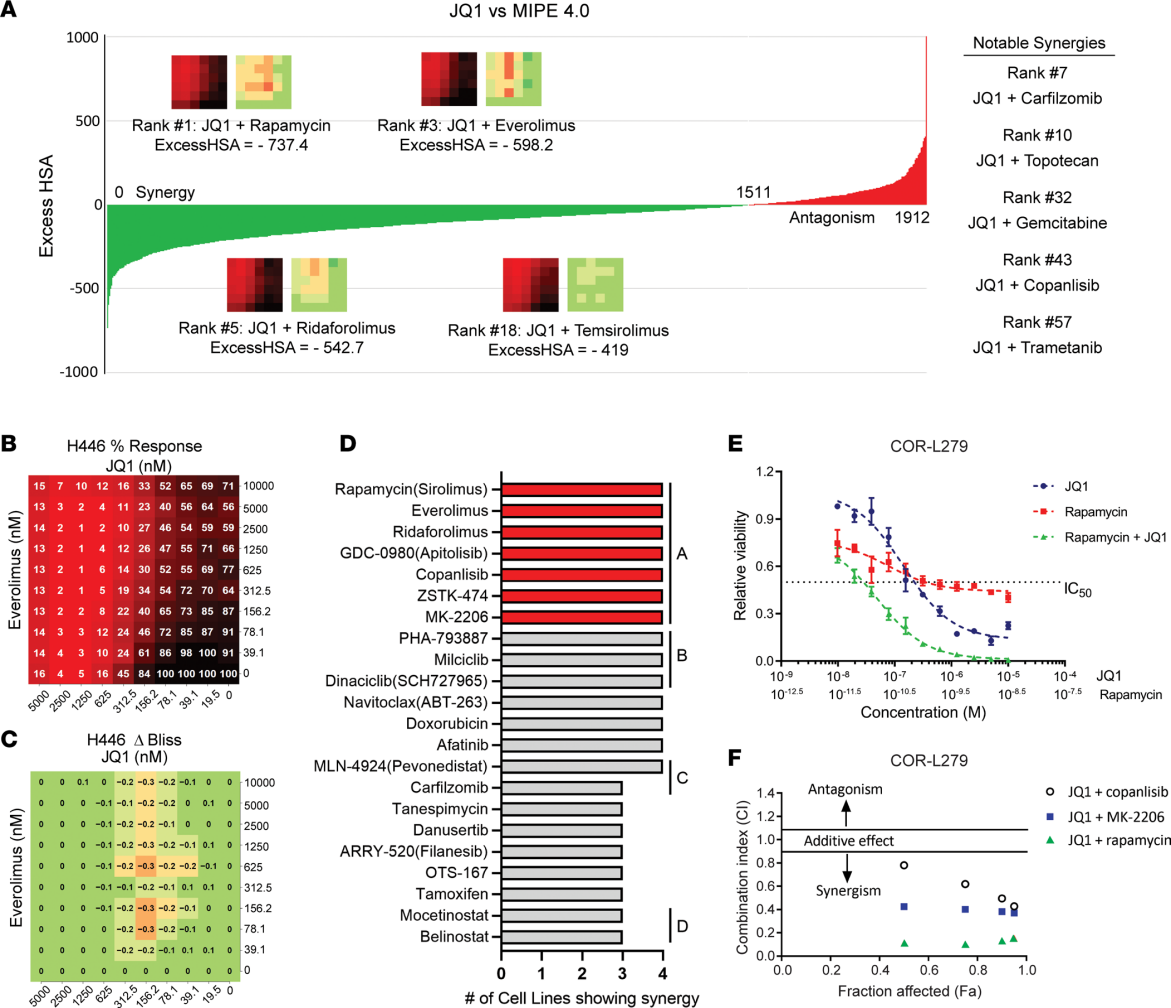
mTORis synergize with BETis in vitro. (**A**) Ranking of synergy and antagonism of JQ1 in combination with 1,912 agents from the MIPE 4.0 library in H446 cells on the basis of the excess HSA metric. Prominent drug synergies, including the combination of JQ1 with several mTORis, are highlighted. (**B** and **C**) The percentage response and Δ Bliss heatmaps of the everolimus/JQ1 combination in a 10 × 10 layout in H446 cells. (**D**) The drugs that synergized with BETis in more than or equal to 3 out of 4 SCLC lines in the 10 × 10 screens. **A**–**D** represent the inhibitors targeting the PI-3K–AKT–mTOR pathway, cyclin-dependent kinases, proteolysis pathway, and histone deacetylases, respectively. (**E**) Relative viability of COR-L279 cells 72 hours after treatment with JQ1, rapamycin, or their combination. Each symbol and error bar show the mean ± SD of 4 replicates. (**F**) Synergy plot showing the CIs versus affected fractions of COR-L279 cells on the basis of the viability results at 72 hours after treatment of specified drug combinations.

We first examined the combination of JQ1 (a prototype BETi) and a Mechanism Interrogation Plate (MIPE) 4.0 library of 1,912 approved and investigational drugs, using a 6 ***×*** 6 matrix layout and an end point of cell viability at 72 hours after treatment. The outcomes of this experiment demonstrated a strong enrichment of mTORi agents (e.g., rapamycin, everolimus, ridaforolimus, and temsirolimus) among the highest-ranked synergetic drug-drug pairs (Figure 1A and Supplemental Table 1; supplemental material available online with this article; https://doi.org/10.1172/jci.insight.156657DS1).

Follow-up studies explored the 40 most promising outcomes using an expanded 10 × 10 matrix layout with optimized concentration ranges. JQ1 and another BETi, I-BET762, were tested in parallel across 4 SCLC cell lines of 2 molecular subtypes (SCLC-N subtype: H446, COR-L279, and SCLC-21H; and SCLC-A subtype: H187). In addition to cell viability at 72 hours after treatment, we also measured apoptosis induction at 8- and 16-hour intervals. Figure 1, B and C, presents an example of drug response and synergy heatmaps, in which JQ1 synergized with everolimus in a broad dose range of 39.1–312.5 nmol/L. No synergy was observed at the JQ1 dose greater than or equal to 625 nmol/L due to a strong single-agent activity (≤ 16% viable). Figure 1D and Supplemental Table 2 list 22 drugs that are synergistic with JQ1 and I-BET762 in more than or equal to 3 out of the 4 SCLC cell lines, with inhibitors targeting the PI-3K–AKT–mTOR pathway at the top of the list.

To validate the results of the high-throughput screens, we performed independent viability assays using a fixed concentration ratio of 2-drug combinations. Figure 1E shows a typical example in COR-L279 cells, in which concurrent treatment of rapamycin and JQ1 decreased the IC_50_ values of both JQ1 and rapamycin by 10 fold or more. We quantified the synergy between JQ1 and the inhibitors targeting PI-3K (copanlisib), AKT (MK-2206), or mTOR (rapamycin) using the method of Chou and Talalay (18). In this method, a smaller combination index (CI) indicates a higher degree of synergy (e.g., CI 0.1–0.3: strong synergism; CI 0.3–0.7: synergism; CI 0.9–1.1: additive; and CI > 1.1: antagonism). On the basis of the CIs at 4 different dose levels that caused a 50%, 75%, 90%, and 95% decrease in cell viability (fraction affected), the combination of JQ1 and rapamycin showed the highest degree of synergy (the smallest CIs) in COR-L279, H446, and H187 cells, especially at the first 2 dose levels (Figure 1F; and Supplemental Figure 1, A and B). To assess the toxicity of this combination in normal human lung cells, we compared the everolimus/JQ1 combination with 2 other synergistic combinations (ABT-263 [an inhibitor of BCL2 and BCL-xL]/JQ1 and copanlisib/JQ1) in the Cdk4/hTERT-immortalized human bronchial epithelial cells (HBEC). When combined with JQ1, everolimus caused a substantially smaller decrease in HBEC’s viability compared with ABT-263 and copanlisib, suggesting the everolimus/JQ1 combination is less toxic to normal lungs (Supplemental Figure 2, A–C). On the basis of these results, we chose to focus on the mTORi/BETi combination for further investigation.

To ensure the synergy between rapamycin and JQ1 was not due to a drug off-target effect, we evaluated the combinations of JQ1 and various mTORis (first-generation: rapamycin and everolimus; and second-generation: torkinib) and the pairing of everolimus with another BETi-NHWD870 (19). Supplemental Figure 3A shows that the concurrent treatment of various mTORis and BETis consistently demonstrated a strong synergy. We also tested the combination of JQ1 and everolimus in SCLC cell lines resistant to JQ1 (IC_50_ ≥ 20 μmol/L), in which a strong synergy was observed in 2 out of 3 cell lines (Supplemental Figure 3B). Collectively, these results demonstrate that mTORis form a strong synergy with BETis in various molecular subtypes of SCLC in vitro.

### mTOR inhibition amplifies BETi-induced apoptosis in SCLC.

**Figure 2:**
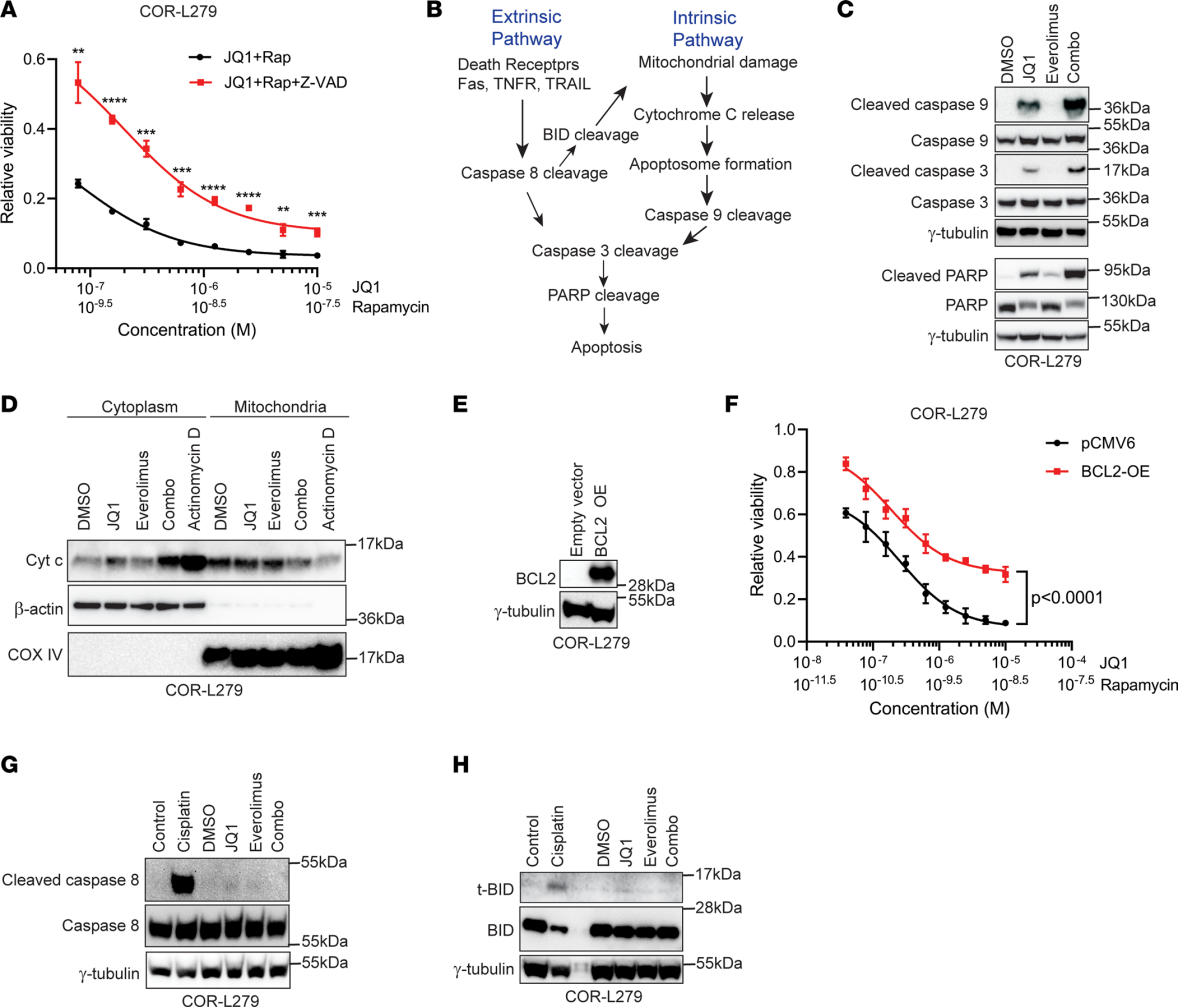
mTOR inhibition amplifies the BETi-induced apoptosis via the intrinsic apoptotic cascade in SCLC. (**A**) A pancaspase inhibitor, Z-VAD-FMK (2.5 μM), ameliorates the growth inhibition caused by the rapamycin/JQ1 combo treatment (72 hours) in COR-L279 cells. (**B**) A diagram illustrating the intrinsic and extrinsic apoptotic cascades. (**C**) Western blots show the cleavage of caspase 9, caspase 3, and PARP in COR-L279 cells following a 24-hour treatment of JQ1 (1 μM), everolimus (6.25 nmol/L), or their combination. The results were obtained from the same biological samples run in 2 gels on the same day. (**D**) Western blots show cytochrome c levels in mitochondrial and cytoplasmic fractions of COR-L279 cells after a 16-hour treatment of JQ1 (1 μM), everolimus (6.25 nmol/L), or their combination. Actinomycin D serves as a positive control, and β-actin and COX IV are loading controls for cytoplasmic and mitochondrial fractions, respectively. (**E**) Western blots show ectopic expression (OE) of BCL2 in COR-L279 cells. (**F**) Effects of ectopic BCL2 on COR-L279 cell viability after a 72-hour treatment of the rapamycin/JQ1 combination. (**G** and **H**) Western blots show cleaved caspase 8 and t-BID in COR-L279 cells treated with JQ1 (1 μM), everolimus (6.25 nmol/L), or their combination. Cisplatin at 20 and 50 μM/ were used as positive controls. The significance of the 2-group comparisons in **A** and **F** was determined using the Student’s *t* test with the FDR multiple comparison corrections, and each symbol and error bar shows the mean ± SD of at least 4 replicates. **P < 0.01; ***P < 0.001; ****P < 0.0001.

To determine whether the mTORi/BETi combinations induce apoptosis in SCLC, we treated COR-L279 cells with the combination of JQ1 and rapamycin in the presence of a pancaspase inhibitor, Z-VAD-FMK. Figure 2A shows that Z-VAD-FMK drastically attenuated the decrease in cell viability caused by the rapamycin/JQ1 combination, suggesting that apoptosis is partly responsible for growth inhibition. Apoptosis can be triggered through either the intrinsic or extrinsic cascade, with the former pathway characterized by the cleavage of caspase 9 and the latter by the cleavage of caspase 8 (Figure 2B). Both cascades converge on the cleavage of caspase 3 and PARP to activate cell death, and crosstalk between these 2 pathways can occur through cleaved BID (also known as t-BID).

To dissect which apoptotic pathway is activated by the mTORi/BETi combination, we first examined cleaved caspase 9, a key intermediate of the intrinsic cascade. Figure 2C shows that JQ1 increased cleaved caspase 9 and the downstream cleaved caspase 3 and PARP, while everolimus induced cleaved PARP in COR-L279 cells. Combining both inhibitors further increased these cleaved apoptotic proteins. These results demonstrate that JQ1 and the combo activate the intrinsic apoptotic pathway. Consistent with the viability results shown earlier, we confirmed that the rapamycin/JQ1 combination caused more apoptosis than JQ1 alone in COR-L279 and H446 but not in COR-L88 cells by using a caspase 3 and 7 activity assay (Supplemental Figure 4).

Because the intrinsic apoptotic cascade is typically triggered by the release of cytochrome c from mitochondria (Figure 2B), we isolated cytosolic and mitochondrial fractions of COR-L279 cells after drug treatments. Figure 2D shows that cytochrome c was elevated in the cytosolic fraction of the JQ1-treated cells and was substantially higher in the sample treated with the everolimus/JQ1 combination, consistent with the activation of the intrinsic apoptotic pathway by BETis and the combo. To further demonstrate the involvement of this cascade, we overexpressed BCL2, an antiapoptotic protein that stabilizes mitochondrial membrane and blocks cytochrome c efflux, in COR-L279 cells (Figure 2E). Ectopic expression of BCL2, which as expected sensitized COR-L279 cells to ABT-199, a BCL2 inhibitor (Supplemental Figure 5), attenuated the growth inhibition induced by the rapamycin/JQ1 combination (Figure 2F).

Next, we assessed whether the everolimus and JQ1 combination activates the extrinsic apoptotic pathway. Figure 2G shows that cisplatin, but not JQ1 or the everolimus/JQ1 combination, induced cleaved caspase 8 in COR-L279 cells. t-BID was elevated in cells treated with cisplatin but not in those receiving JQ1 or the drug combo (Figure 2H). Together, these results demonstrate that the mTORi and BETi combination induces apoptosis by activating the intrinsic apoptotic pathway in SCLC.

### mTOR inhibition augments the antitumor effects of BETis in vivo.

**Figure 3:**
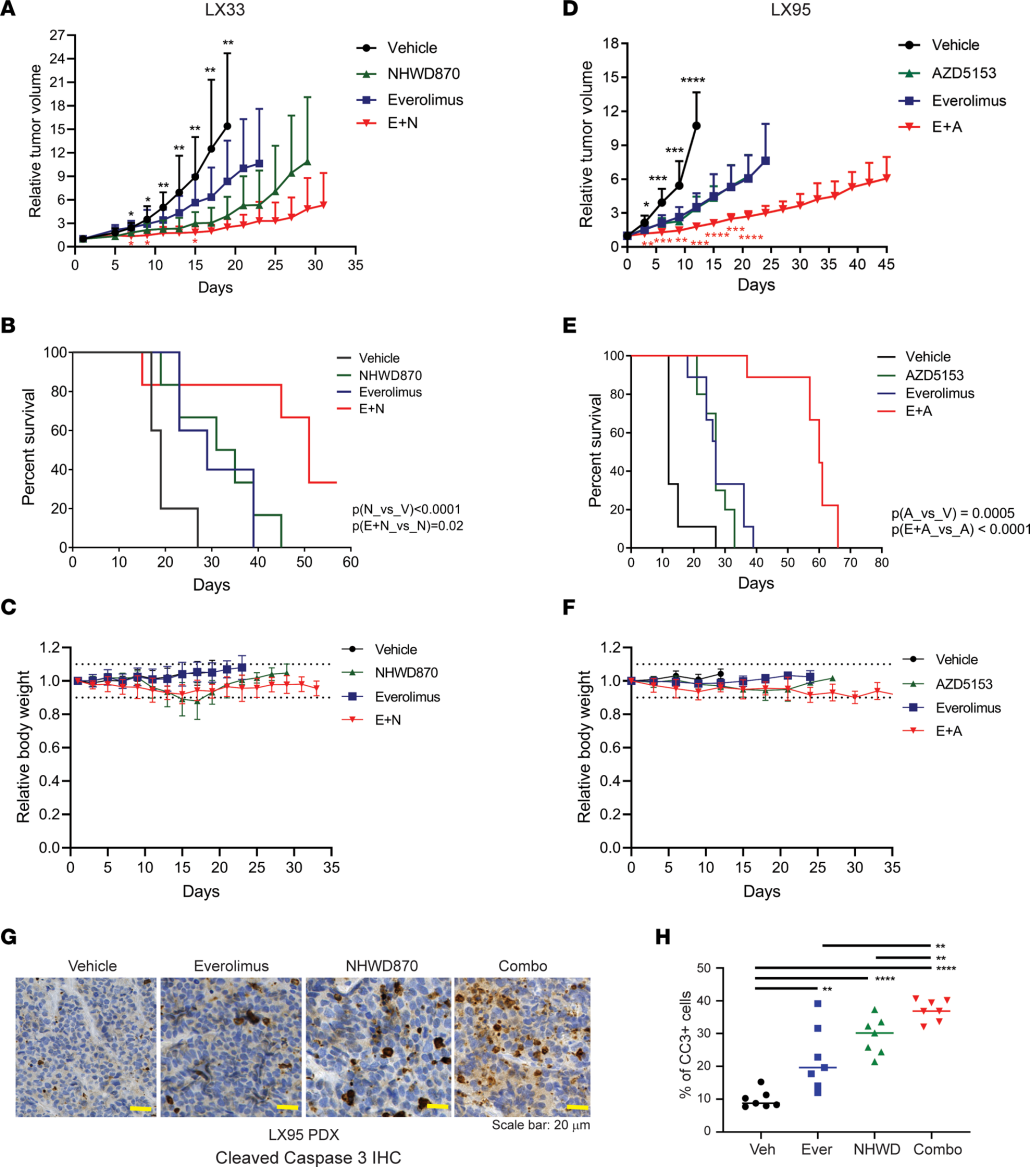
mTOR inhibition augments the antitumor effects of BETis by increasing apoptosis in vivo. (**A** and **B**) The combination of everolimus (3 mg/kg, daily) and NHWD870 (3 mg/kg, daily) was more effective than single agents in controlling tumor growth as shown in A and prolonging survival as shown in B in LX33, an SCLC-N PDX model (*n* ≥ 10 per group). (**C**) Relative BW changes of the mice in **A**. (**D** and **E**). The combination of everolimus and AZD5153 (both at 1 mg/kg, daily) was more effective than single agents in controlling tumor growth as shown in **D** and prolonging survival as shown in **E** in LX95, an SCLC-A PDX model (*n* = 9 per group). (**F**) Relative BW changes of the mice in **D**. (**G** and **H**) Representative images and quantification of cleaved caspase 3 IHC staining in LX95 PDX tumors after a 1-week treatment of everolimus (2 mg/kg, daily), NHWD870 (1.5 mg/kg, daily), or the combination (everolimus 1.5 mg/kg and NHWD870 1 mg/kg, daily). *n* = 7 per group. Horizontal lines in **H** represent medians. The significance of the 2-group comparisons was determined using the Student’s *t* test with the FDR multiple comparison corrections in **A** and **D**, the log-rank test in **B** and **E**, and the ANOVA test with Dunnett’s multiple test corrections in **H**. Statistically significant differences in tumor volumes between the BETi single-agent and vehicle groups are indicated with black asterisks, while red asterisks mark the statistically significant changes between the combo and the BETi single-agent groups in **A** and **D**. Dotted lines represent 10% weight gain or loss in **C** and **F**. Data are shown as mean ± SD; *P < 0.05; **P < 0.01; ***P < 0.001; ****P < 0.0001. A, AZD5153; E, everolimus; N, NHWD870; V, vehicle; CC3, cleaved caspase 3. Scale bar: 20 µm.

To assess whether mTORis potentiate the antitumor effects of BETis in vivo, we chose NHWD870 over JQ1 because the latter has unfavorable pharmacokinetic properties in vivo (20). We first tested the combination of everolimus and NHWD870 in an SCLC-N subtype patient-derived xenograft (PDX) model — LX33. Figure 3, A and B, shows that concurrent administration of everolimus and NHWD870 resulted in significantly better control of tumor growth and a longer median overall survival than single drugs (50 days in the combo group versus 30 days in single-agent groups). This combination was reasonably tolerated except for mild diarrhea and weight loss (Figure 3C), which typically improved spontaneously after pausing the treatments for a few days.

To confirm that the antitumor activity of the mTORi/BETi combo is not restricted to a specific drug or a molecular subtype of SCLC in vivo, we tested AZD5153, a bivalent BETi in phase I/II clinical trials (21), in an SCLC-A subtype PDX — LX95. Figure 3, D and E, shows that, compared with single agents, concurrent administration of everolimus and AZD5153 achieved significantly better control of LX95 tumor growth and increased the median overall survival (30 days in single-agent groups versus 60 days in the combo group). The combination of everolimus and AZD5153 was well tolerated without causing substantial weight loss or diarrhea (Figure 3F). To test whether AZD5153 has antitumor activity at clinically achievable dose levels, we established a tumor cell line from the LX95 PDX. Supplemental Figure 6A shows that the histology of the xenograft tumors formed from the injected cell line was identical to LX95 PDX tumors. AZD5153 in the dose ranges of 80–200 nmol/L, which corresponds to the highest plasma concentrations (Cmax) in the patients treated at 10–40 mg daily (22), effectively suppressed the growth of LX95 and COR-L279 by 45–55% and 30–45%, respectively (Supplemental Figure 6B).

To assess whether the antitumor effect persists after stopping the drug combination, we measured the tumor growth of LX95 PDX after a short pause of the treatments. Supplemental Figure 7 shows that the tumors in the NHWD870 or everolimus single-agent groups resumed growth after holding treatment for 3 days, while those in the combo group remained the same size. To determine whether the persistent antitumor effect of the combo is due to an increase in apoptosis, we treated the LX95 models with the everolimus and NHWD870 combination for 1 week. Like the everolimus/AZD5153 combination, the everolimus/NHWD870 combo resulted in better tumor control than single agents without causing significant weight loss in this model (Supplemental Figure 8, A and B). More cleaved caspase 3-positive tumor cells were observed in the combo group than the single-agent groups, suggesting that increased apoptosis contributes to the persistent antitumor effects of the combo (Figure 3, G and H).

To determine whether the increased apoptosis was mediated through activation of the intrinsic apoptotic pathway, we treated LX95 cells with the everolimus and JQ1 combination in vitro. Like COR-L279, JQ1 increased cleaved caspase 3 and 9 and cleaved PARP, while everolimus induced cleaved caspase 3 and cleaved PARP in LX95 (Supplemental Figure 9A). The combination resulted in more cleaved apoptotic proteins and cytochrome c release from mitochondria than the single drugs, consistent with activation of the intrinsic apoptotic pathway (Supplemental Figure 9, A and B). Unlike COR-L279, caspase 8 was barely detectable in LX95 cells (Supplemental Figure 9C), reflecting the frequent loss of this gene in SCLC (23). This result essentially rules out the involvement of the extrinsic apoptotic cascade in the synergy of the mTORi/BETi combo in the LX95 model.

We next asked how the mTORi and BETi combination compares to other drug pairings in vivo. To this end, we evaluated the combination of AKT inhibitors (AKTis) and BETis in the LX95 model. Compared with the AZD5153 alone, the combination of AZD5363 (an AKTi) and AZD5153 did not result in better control of tumor growth or a longer median overall survival (Supplemental Figure 10, A and B). This lack of improvement was due to excessive toxicity, as this combination caused severe weight loss, diarrhea, and premature death, none of which occurred in single-agent groups. Despite a 40% dose reduction of AZD5363 2 weeks after the first dose, this combination was still challenging to administer and necessitated prolonged treatment holding. Collectively, these results demonstrated that the mTORi/BETi combination results in superior antitumor effects than single drugs or the pairing of AKTi/BETi in vivo.

### BETi upregulates RSK3, a potential upstream kinase of the TSC2-mTOR cascade, in SCLC.

BETis have been reported to synergize with mTORis by blocking the mTORi-induced RTK feedback activation in breast and colon cancer cell lines (16, 17). To determine whether this feedback mechanism explains the synergy between mTORis and BETis in SCLC, we compared the effects of mTOR inhibition on AKT T308 phosphorylation (an indicator of RTK feedback activation; ref. 14–17) between breast cancer (MDA-MB-468) and SCLC (COR-L279 and LX95) cell lines. mTOR inhibition resulted in a steady increase in AKT T308 phosphorylation only in MDA-MB-468 cells but not in COR-L279 and LX95 cells, suggesting that a different mechanism is responsible for the drug synergy in SCLC (Supplemental Figure 11, A–E).

**Figure 4:**
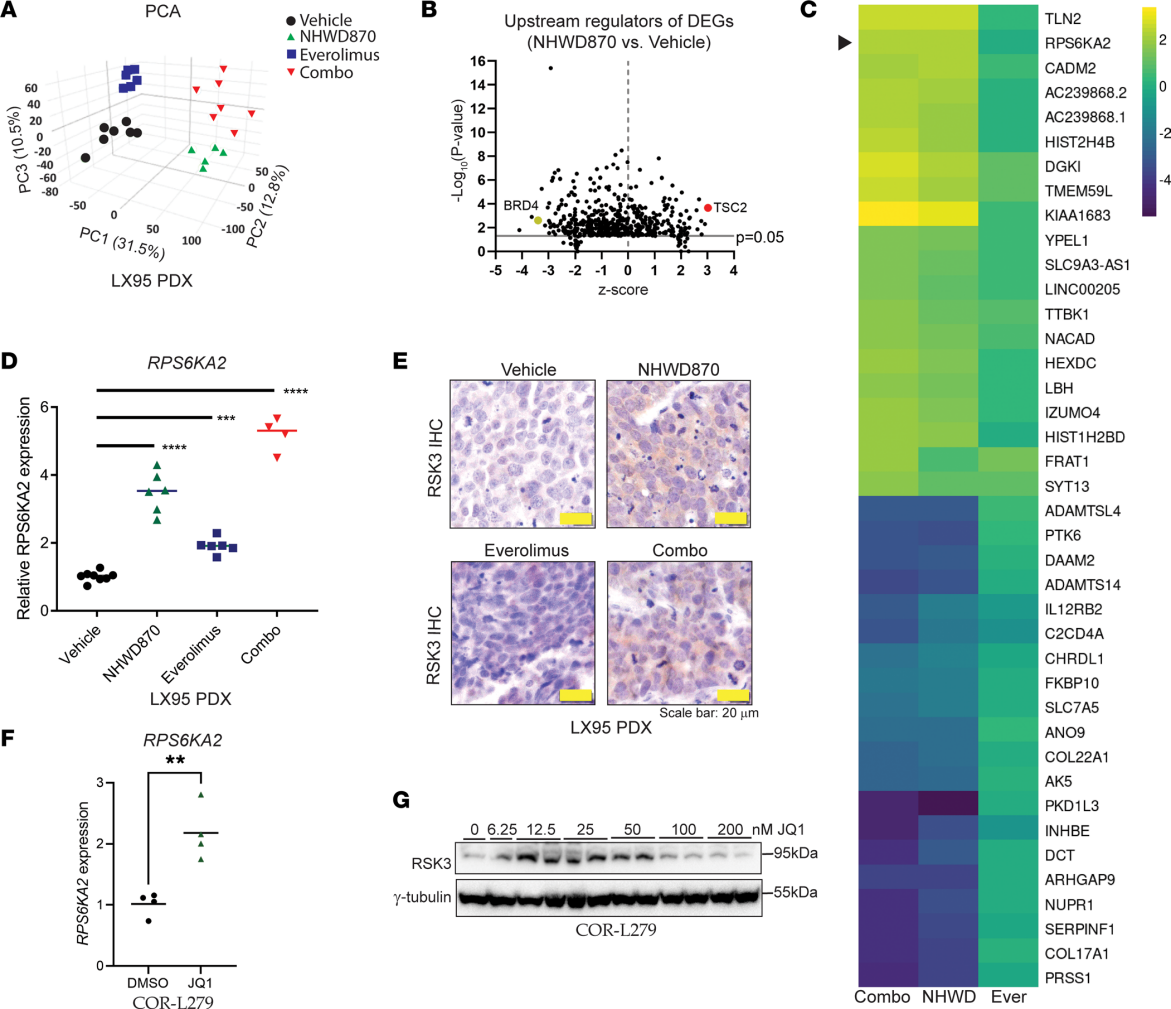
BETi upregulates *RPS6KA2* and its encoded protein RSK3 in SCLC in vitro and in vivo. (**A**) Principal Component Analysis plot showing clustering of the RNA-Seq transcriptomes of LX95 PDXs in the experiment described in Figure 3H. Due to insufficient RNA, 2 tumors from the NHWD870 group and 1 from the Everolimus group were not profiled. (**B**) Volcano plot showing the predicted upstream regulators on the basis of the significant genes (>2-fold change and adjusted *P* < 0.05) in the tumors treated with NHWD870 versus Vehicle in **A**. The *y* axis displays the statistical significance and the *x* axis shows the degree of activation/inhibition (*Z* score). (**C**) Heatmap of the top 20 up- and downregulated genes in the Combo group relative to the Vehicle group. The arrowhead points to *RPS6KA2*. (**D** and **E**) qPCR and IHC staining assessing expression of *RPS6KA2* gene and RSK3 protein in the LX95 PDX tumors in **A**, respectively. (**F** and **G**) *RPS6KA2* gene and RSK3 protein expression in COR-L279 cells following 1-week treatment of JQ1 at 50 nmol/L in **F** or at the specified doses in **G**. Fresh media and JQ1 were replenished every 3 days. Two experimental replicates were used for JQ1 doses greater than or equal to 12.5 nmol/L. The significance of the 2-group comparisons was determined using the ANOVA test with Dunnett’s multiple test corrections in **D** and the Student’s *t* test in **F**. **P < 0.01; ***P < 0.001; ****P < 0.0001. Scale bar: 20 µm.

To identify the underlying mechanism of drug synergy, we performed RNA-Seq to profile LX95 PDXs following a 1-week treatment of everolimus, NHWD870, or the combined inhibitors. Principal-component analysis showed that the transcriptomes of the tumor cells were clustered into 4 treatment groups (Figure 4A). A total of 929 genes had at least a 2-fold change with an adjusted *P* value less than 0.05 when comparing the NHWD870-treated tumors to those receiving the vehicle (Supplemental Table 3). Ingenuity Pathway Analysis of the identified genes correctly predicted that BRD4 was inactivated by NHWD870, while TSC2, an upstream regulator of mTOR signaling, was among the top activated regulators (Figure 4B). Interestingly, one of the 20 most upregulated genes in the NHWD870 and the combo groups was *RPS6KA2* (Figure 4C), which belongs to the same family of *RPS6KA1 —* a known upstream kinase of TSC2 (24). Using quantitative PCR (qPCR) and IHC staining, we confirmed that *RPS6KA2* gene and its protein, RSK3, were induced by NHWD870 and the combo in LX95 tumors (Figure 4, D and E).

We modeled the in vivo experiment by treating COR-L279 cells with various doses of JQ1 for 1 week in vitro. JQ1 was confirmed to upregulate *RPS6KA2* and RSK3 at multiple tested concentrations (Figure 4, F and G). Consistent with the results shown earlier, a similar degree of *RPS6KA2* induction was observed following JQ1 treatment in LX95 but not in COR-L88 cells (Supplemental Figure 12). Together, these results show that BETi induces RSK3, a potential upstream kinase of TSC2, in SCLCs that respond synergistically to the mTORi/BETi combination.

### RSK3 induction increases resistance to BETi-induced apoptosis.

**Figure 5:**
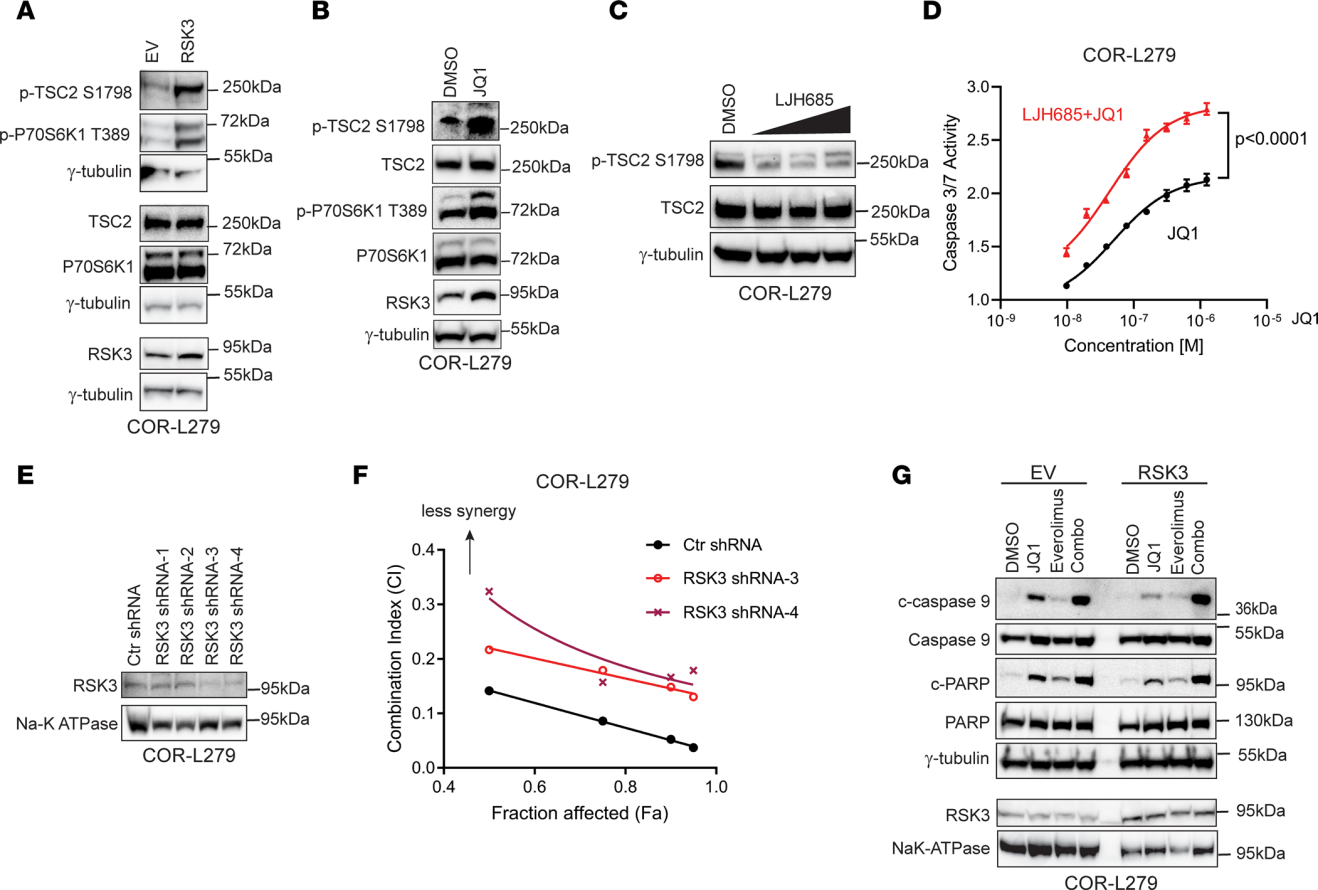
RSK3 upregulation attenuates the BETi-induced apoptosis. (**A**) Overexpression of Myr-RSK3 increases phosphorylation at TSC2 S1798 and p70S6K1 T389 in COR-L279 cells. The results were obtained from the same biological samples run on 3 gels on different days. (**B**) JQ1 treatment (50 nmol/L, 7 days) induced the phosphorylation at TSC2 S1798 and p70S6K1 T389 in COR-L279 cells. (**C**) Reduction of TSC2 S1798 phosphorylation after LJH685 treatment at 5, 10, or 20 μM for 24 hours. (**D**) LJH685 (10 μM) augmented the JQ1-induced caspase 3/7 activation in COR-L279 cells. The significance of the 2-group comparisons was determined using the Student’s *t* test with the FDR multiple comparison corrections. The data are shown as mean ± SD of 4 replicates. (**E**) Western blot showing RSK3 KD by 4 shRNAs in COR-L279 cells. NaK-ATPase serves as a loading control for membrane fraction proteins. (**F**) The synergy plot shows that RSK3 KD attenuated the synergy of the rapamycin/JQ1 combination in COR-L279 cells. (**G**) Overexpression of Myr-RSK3 attenuated apoptosis in COR-L279 cells treated with JQ1 but not in those receiving the everolimus and JQ1 combination. One day after transient transfection of Myr-RSK3 or EV, COR-L279 cells were treated with JQ1 (1 μM), everolimus (10 nmol/L), or their combination for 24 hours before measuring cleaved PARP and caspase 9. The results on RSK3 and its loading control NaK-ATPase were from a repeat experiment.

To evaluate the functional significance of RSK3 induction, we overexpressed a myristoylated (myr) RSK3 in COR-L279 cells. The myr sequence recruits overexpressed RSK3 to the cell membrane, causing activation of its kinase activity (25). Compared with an empty vector (EV), forced expression of myr-RSK3 increased phosphorylation at TSC2 S1798, demonstrating that TSC2 is a kinase substrate of RSK3 (Figure 5A). Phosphorylation at p70S6K1 T389, a direct downstream target of mTORC1, was also elevated in the RSK3-overexpressed cells, indicating that RSK3 augments mTOR signaling (Figure 5A). To determine whether BETi-induced RSK3 could activate the TSC2-mTOR-p70S6K1 signaling, we treated COR-L279 cells with JQ1 for 1 week and observed an increase in both TSC2 S1798 and p70S6K1 T389 phosphorylation (Figure 5B). The increase in phosphorylation of these 2 kinases started as early as 24 and 48 hours following JQ1 treatment and persisted in a 6-day treatment of COR-L279 cells (Supplemental Figure 13A; and the red curves in Supplemental Figure 13, C and D). In contrast, phosphorylated TSC2 S1798 and p70S6K1 T389 remained unchanged in COR-L88 cells at most of the examined intervals (Supplemental Figure 13B; and the black curves in Supplemental Figure 13, C and D). Together, these results suggest that the activation of the TSC2-mTOR-p70SK1 cascade plays a role in BETi sensitivity in SCLC.

To evaluate how RSK3 upregulation affects the BETi sensitivity in SCLC, we first used LJH685, a selective inhibitor of the RSK family kinases (26), to block TSC2 phosphorylation induced by RSK kinases (Figure 5C). Figure 5D shows that LJH685 augmented caspase 3/7 activation following JQ1 treatment in COR-L279 cells. Because LJH685 inhibits all members of the RSK family, we used shRNA to knockdown (KD) RSK3. Figure 5E shows that the stable transfection of 2 (3 and 4) out of 4 shRNAs in COR-L279 cells led to a substantially reduced expression of RSK3. RSK3 KD diminished the synergy of the mTORi/BETi combination in COR-L279 cells, confirming that RSK3 upregulation plays a role in the synergy (Figure 5F). To assess the roles of other RSK family kinases, we used shRNA to KD RSK1 and RSK2 in COR-L279 cells (Supplemental Figure 14A). The KD of RSK1, but not RSK2, modestly decreased the synergy of BETis and mTORis (Supplemental Figure 14B). To assess if the effects of RSK1 KD and RSK3 KD are interconnected, we evaluated the induction of *RPS6KA2* gene after RSK1 KD. Supplemental Figure 14C shows that RSK1 KD lessened the BETi-induced *RPS6KA2* upregulation, suggesting that the effect of RSK1 KD on the synergy is mediated through RSK3.

To further confirm that RSK3 is responsible for BETi resistance, we overexpressed myr-RSK3 in COR-L279 cells and observed a decrease in the JQ1-induced caspase 9 and PARP cleavage, suggesting that RSK3 induction attenuates the JQ1-induced apoptosis (Figure 5G). Everolimus abrogated the protective effect of RSK3 overexpression, as the combo induced the same level of cleaved caspase 9 and PARP in RSK3-overexpressed cells as the control (Figure 5G). Collectively, these findings demonstrate that BETi-induced RSK3 attenuates BETi-induced apoptosis by activating the TSC2-mTOR signaling and combining mTORi abolishes such a protective mechanism.

### p70S6K1 mediates the antiapoptotic effects of RSK3 by phosphorylating BAD.

**Figure 6:**
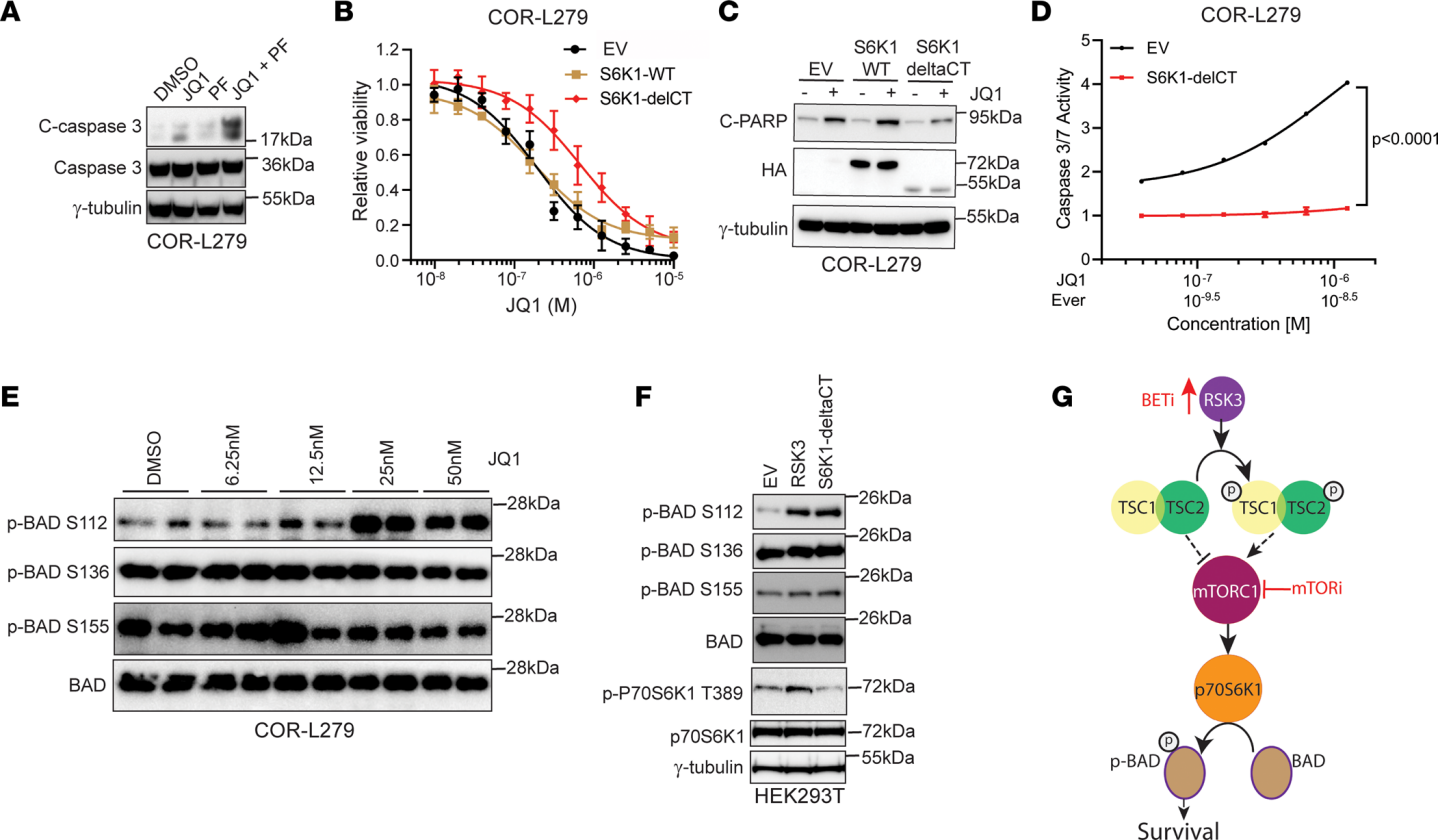
p70S6K1 mediates the antiapoptotic effects of RSK3. (**A**) Cleavage of caspase 3 in COR-L279 cells at 24 hours after treatment with JQ1 (1.67 μM), PF-4708671 (PF; 16 μM), or their combination. (**B** and **C**) Overexpression of an active form of rat S6K1 (HA-S6K1-ΔCT), but not its WT (HA-S6K1-WT), attenuated the JQ1-induced growth inhibition and apoptosis in COR-L279 cells. JQ1 was administered at the specified doses for 72 hours 2 days after transfection as shown in **B** or at 1 μM for 24 hours as shown in **C**. (**D**) Overexpression of S6K1-ΔCT attenuated the apoptosis induced by the everolimus and JQ1 combination. Two days after transfection with S6K1-ΔCT or EV, COR-L279 cells were treated with the JQ1 and everolimus at a fixed ratio for another 48 hours before the caspase 3/7 activity was measured. The significance of the 2-group comparisons was determined using the Student’s *t* test with the FDR multiple comparison corrections. (**E**) Western blots show changes in BAD phosphorylation at S112, S136, and S155 after 1-week JQ1 treatment. (**F**) Effects of RSK3 and S6K1-ΔCT overexpression on BAD phosphorylation in HEK293T cells. (**G**) A proposed model shows that BETi induces RSK3 in SCLC, which activates the TSC2-mTOR-p70S6K1-BAD pathway to increase cell survival, and mTORi blocks this protective signaling and augments BETi-induced apoptosis. Error bar represents SD of at least 4 replicates in **B** and **D**. The results in **E** and **F** were obtained from the same biological samples run in separate gels in parallel on the same day.

To determine whether p70S6K1 mediates the antiapoptotic effects of RSK3, we treated COR-L279 cells with JQ1 and PF-4708671, a specific inhibitor of p70S6K1. As shown in Figure 6A, the PF-4708671/JQ1 drug pairing recapitulated the increased apoptosis caused by the mTORi/BETi combinations. To further define the role of p70S6K1, we overexpressed a constitutively active form of rat S6K1 (S6K1-ΔCT) and its WT (S6K1-WT) in COR-L279 cells. As shown in Figure 6B, S6K1-ΔCT, but not S6K1-WT, increased the viability of cells treated with JQ1. For example, at the JQ1 dose of 0.5 μM, the viability of the cells with control, S6K1-WT, and S6K1-ΔCT vectors were 25%, 30%, and 55%, respectively. Consistent with these findings, overexpression of S6K1-ΔCT, but not S6K1-WT, attenuated the induction of cleaved PARP by BETi (Figure 6C). Furthermore, overexpression of S6K1-ΔCT abrogated the apoptosis induced by the drug combination (Figure 6D). Together, these results indicate that p70S6K1 is the primary downstream kinase of mTOR that mediates the synergy of the drug combo.

To identify the downstream effector of p70S6K1, we examined the phosphorylation of the proapoptotic protein BAD, a known substrate of p70S6K1 (27). Phosphorylation of BAD releases antiapoptotic proteins BCL-2 and BCL-xl and promotes survival (28). After JQ1 treatment for 1 week, phosphorylation at BAD S112, but not at S136 and S155, increased in COR-L279 cells (Figure 6E). A similar change in the BAD phosphorylation profile was observed after overexpression of RSK3 or S6K1-ΔCT in HEK293T cells, suggesting that RSK3 increases BAD phosphorylation via p70S6K1 (Figure 6F).

We next assessed whether autophagy, a downstream pathway suppressed by mTOR, plays a role in the synergy between mTORis and BETis. Consistent with the known autophagy induction after mTOR inhibition, LC3B-II, a marker of autophagosome formation (29), was modestly induced by everolimus (Supplemental Figure 15). JQ1 did not induce LC3B-II nor affect everolimus-induced LC3B-II, suggesting that altered autophagy is not responsible for the synergy between mTORis and BETis (Supplemental Figure 15).

To ensure that our findings on the activation of the RSK3-TSC2-mTOR-p70S6K1-BAD cascade by BETi are not restricted to 1 molecular subtype of SCLC, we treated LX95 cells with JQ1 for 1 week. Like COR-L279, JQ1 increased RSK3 and phosphorylation at TSC2 S1798, p70S6K1 T389, and BCL2 S112 at multiple dose levels in LX95 cells (Supplemental Figure 16). Together, our findings support a model that RSK3 increases BAD phosphorylation via the TSC2-mTOR-p70S6K1 signaling cascade to promote survival, and mTORi blocks this signaling and potentiates BETi-induced apoptosis (Figure 6G).

## Discussion

Multiple investigational BETi drugs have been evaluated in early-phase clinical trials, and the results generally showed modest response rates and a short duration of response (30). Rational drug combinations could augment BETi antitumor efficacy and delay drug resistance development (30). Here, we demonstrate that mTORis potentiate BETi antitumor effects in SCLC. We identified RSK3 induction as a potentially novel resistance mechanism to BETis in SCLC, and mTORis augment the BETi-induced apoptosis by blocking the downstream signaling of this kinase.

Using high-throughput combination screens, we identified several classes of drugs synergizing with BETis in an unbiased manner. Some of these candidates, such as BCL2 and HDAC inhibitors, have been shown to potentiate the antitumor effects of BETis in SCLC (8, 11). Several others, such as carfilzomib and inhibitors of cyclin-dependent kinases, had synergy with BETis in other tumor types (31, 32). These studies indirectly validate our results, suggesting shared vulnerabilities between various tumor types.

We focused on the mTORi and BETi combination for further validation and mechanistic studies. We demonstrate that this combination has better antitumor effects than the single agents in vitro and in vivo. In addition, our results suggest that the synergy of the mTORi/BETi combination is not restricted to the SCLC-N subtype or BETi-sensitive SCLC. Finally, this combo was relatively well-tolerated, likely because the adverse effects of mTORis and BETis affect different organ systems. In contrast, the AKTi and BETi combo had poor tolerability in vivo because of overlapping toxicities (e.g., diarrhea) of AKTis and BETis (22, 33). These results stress the need to select drugs with nonoverlapping toxicities to increase antitumor activity and minimize toxicities.

It was previously reported in various tumors that BETis induce apoptosis by decreasing the expression of the subunits in 4 oxidative phosphorylation complexes (34, 35). Consistent with these reports, we found that BETis activate apoptosis through a mitochondria-mediated intrinsic apoptotic cascade, and BCL2 overexpression diminished the apoptosis induced by the mTORi/BETi combination. These results suggest that the mechanism of action underlying the synergy of mTORis and BETis involves the molecular events upstream of mitochondrial apoptosis.

Our results did not validate the feedback RTK activation in SCLC after mTOR inhibition. Previous studies have indicated that EGFR and IGF-1R signaling pathways are commonly involved in this feedback mechanism (15, 17). In SCLC, EGFR signaling is frequently inactive (36), while IGF-1R is upregulated in about 18.5% of primary tumors (37). We speculate that the lack of RTK feedback activation following mTOR inhibition is due to suppressed EGFR signaling in SCLC.

Our study identified *RPS6KA2* induction as a novel mechanism of BETi resistance in SCLC. The prosurvival function of RSK3 is mediated through mTOR signaling, which explains why mTORis would potentiate the antitumor effects of BETis. In breast and ovarian cancers, a higher expression of *RPS6KA2* has been associated with a worse prognosis, suggesting that this gene functions as an oncogene (38, 39). In addition to TSC2, RSK3 can also phosphorylate IκBα and causes activation of NF-κB (39). The mechanism by which BETis increased the expression of *RPS6KA2* remains unclear. Our results suggest that BETis induce *RPS6KA2* through other gene(s), probably RSK1. Consistent with this notion, Tai et al. recently reported that BETis upregulate *RPS6KA2* in breast cancer cell lines by suppressing the BRD4/FOXD3/miR-548d-3p/JunD axis (40). However, we did not find this pathway responsible for the induction of *RPS6KA2* by BETis in SCLC (data not shown), and the underlying mechanism awaits future investigation. Previous studies in other neoplasms have found that BETi resistance involves WNT signaling upregulation via transcriptional program reset (41) or PI-3K–ERK activation through RTK kinome rewiring (42). Our findings reveal a new player — RSK3 — in already diverse mechanisms of BETi resistance and confirm the central role of PI-3K–AKT–mTOR signaling activation.

In conclusion, we demonstrate that mTORi potentiates the antitumor effects of BETis in SCLC by blocking an RSK3-mediated survival signaling cascade. Our findings warrant further evaluation of the mTORi and BETi combination in patients with SCLC.

## Methods

### Cell culture.

H446, H187, H460, H1048, H1436, Calu-6, MDA-MB-468, and HEK293T cells were purchased from the American Type Culture Collection, SCLC-21H was from the German Collection of Microorganisms and Cell Cultures, and COR-L279 and COR-L88 were purchased from Sigma-Aldrich. All commercial cell lines were grown in culture media recommended by the suppliers and were maintained in humidified incubators at 37^o^C. MDA-MB-468 was maintained at 100% air, while all other lines were at 5% CO_2_. Short tandem repeat analysis was performed to authenticate commercial cell lines.

The HBEC cells were provided by John Minna (University of Texas Southwestern, Dallas, Texas) and cultured in Keratinocyte serum-free media (SFM) supplemented with 5 mg/L epidermal growth factor and 50 mg/L bovine pituitary extract (Thermo Fisher Scientific). The LX95 SCLC line was established by growing the single cells isolated from the LX95 PDX tumors in HITES medium (DMEM: F12 medium supplemented with 0.005 mg/mL insulin, 0.01 mg/mL transferrin, 30 nmol/L sodium selenite, 10 nmol/L hydrocortisone, 10 nmol/L beta-estradiol, 1***×*** Glutamax, 5% heat-inactivated FBS, and 1***×*** penicillin/streptomycin). COR-L279 cells with ectopic expression of BCL2 received Flag-BCL2 (a gift from Clark Distelhorst, Case Western Reserve University, Cleveland, OH; ref. 43) (Addgene plasmid 18003) by lipofection followed by 1.0 mg/mL G418 selection (Roche). Stable transfectants of COR-L279 cells with RSK1-shRNA (Sigma-Aldrich; clones TRCN0000001384 and TRCN0000001385), RSK2-shRNA (Sigma-Aldrich; clones TRCN0000194851 and TRCN0000040147), or RSK3-shRNA (Sigma-Aldrich; clones TRCN0000006352, TRCN0000006353, TRCN0000006354, and TRCN0000011010) were selected with 6 μg/mL puromycin (Gibco) after lipofection. All cell lines tested negative for mycoplasma.

### Reagents.

For in vitro studies, ABT-199 (Chemitek, catalog CT-A199), ABT-263 (Chemitek, catalog CT-A263), actinomycin D (Sigma-Aldrich, catalog A9415), AZD5153 (Chemitek, catalog CT-A5153), everolimus (Sellekchem, catalog S1120), (±)-JQ1 (Sigma-Aldrich, catalog SML0974), LJH685 (Sellekchem, catalog S7870), MK-2206 (Sellekchem, catalog S1078), NHWD870 (a gift from Nenghui Wang and Mingzhu Yin, Ningbo Wenda Pharma, Ningbo, China), PF-4708671 (Sellekchem, catalog S2163), rapamycin (Sellekchem, catalog S1039), torkinib (Sellekchem, catalog S2218), and Z-VAD-FMK (Sigma-Aldrich, catalog 219007) were dissolved in DMSO; and copanlisib (Sellekchem, catalog S2802) was prepared in 10% trifluoroacetic acid (Sigma-Aldrich, catalog T6508).

For in vivo studies, AZD5153, AZD5363 (Chemitek, catalog CT-A5363), and NHWD870 were first dissolved in N,N-dimethylacetamide (Sigma-Aldrich, catalog 270555) and then mixed with 0.5% methylcellulose (Sigma-Aldrich, catalog M0430) plus 0.1% Tween-80 (Sigma-Aldrich, catalog P4780) before administration. Everolimus was first dissolved in propylene glycol (Sigma-Aldrich, catalog W294004) at 10 mg/600 μL concentration and was then mixed with 1 mL 10% Tween 80 and finally with 400 μL of water. After mixing, the drug solution was mixed with 0.5% methylcellulose plus 0.1% Tween 80 before each dosing.

The Myr-RSK3 vector (pWZL Neo Myr Flag RPS6KA2; Addgene plasmid 20621) was a gift from William Hahn and Jean Zhao (Dana-Farber Cancer Institute, Boston, MA; ref. 25); the rat S6K1 WT and Δ CT vectors (pRK7-HA-S6K1-E389-ΔCT, Addgene plasmid 8993; and pRK7-HA-S6K1-WT, Addgene plasmid 984) were gifts from John Blenis (Weill Cornell Medicine, New York, NY; ref. 44). These vectors were transfected into cells using either Lipofectamine 2,000 or 3,000 reagents (Thermo Fisher Scientific).

### High-throughput combinatorial screens.

High-throughput combinatorial screens were performed as previously described (45) with some modifications. The initial screen in H446 cells was performed in a 6 ***×*** 6 matrix layout by combining JQ1 with a MIPE 4.0 library, which consists of 1,912 FDA-approved oncological therapeutics and investigational agents. The viability endpoint of the initial screen was measured using the CellTiterGlo reagent (Promega) 72 hours after drug treatment. The synergy of various drug combinations was assessed using the excess Highest Single Agent (HSA) method, the response heat map, and the Δ Bliss heat map (46, 47).

The second screen was performed in 4 SCLC cell lines of 2 molecular subtypes (SCLC-N: H446, COR-L279, and SCLC-21H; and SCLC-A: H187) in a 10 × 10 matrix by combining JQ1 or iBET-762 with 1 of the forty drugs selected from the first screen. The endpoints of the second screen were caspase 3/7 activity measured using the CaspaseGlo 3/7 assay (Promega) at 8-hour and 16-hour intervals, as well as cell viability measured using the CellTiterGlo reagent at the 72-hour interval.

### Cell viability and caspase 3/7 activity assay.

Cells were dissociated with TrypLE (Thermo Fisher Scientific), and large cell clumps were removed by passing the cell solution through sterile 40 μm nylon mesh cell strainers (Thermo Fisher Scientific). Subsequently, 750 cells in 15 μL growth media were seeded into each well of a 384-well plate (Corning, catalog 3765). The next day, drugs were serially diluted 2 fold in appropriate solvents to generate 9 consecutive concentrations. Then, the compound and its vehicle control were diluted 100 times in culture media and delivered to the cells in 10% of the final volume to achieve a total of 1,000 times dilution. Assays were performed with 4 replicates for each dose. Caspase 3/7 activity was measured using the Caspase-Glo 3/7 assay system (Promega) at the 48-hour interval, and cell viability was examined at the 72-hour interval using the CellTiter-Glo 2.0 Reagent (Promega). A reflective foil seal (Bio-Rad, catalog MSF1001) was applied to the bottom of each plate to maximize the output signal.

### Western blot analysis.

After cells were collected and washed with PBS, cells were lysed in cold 1 × RIPA lysis buffer (Millipore) supplemented with a protease inhibitor cocktail (Sigma-Aldrich). When a phosphorylated protein was measured, RIPA lysis buffer was additionally supplied with a phosphatase inhibitor cocktail (Sigma-Aldrich or Thermo Fisher Scientific). After incubating on ice for 10 minutes, cells were centrifuged at 10,000*g* for 10 minutes to extract cell lysates. Mitochondrial fractions (for cytochrome c measurement) and membrane fractions (for Myr-RSK3 measurement) were extracted from cells using a Cell Fractionation kit (Abcam) and a Mem-PER Plus Membrane Protein Extraction Kit (Thermo Fisher Scientific), respectively.

Proteins were quantified using a Pierce BCA Protein Assay Kit (Thermo Fisher Scientific). Equivalent amounts of protein were resolved on precast polyacrylamide denaturing gels and were then transferred onto 0.2 μm PVDF membranes using either dry transfer with an iBLOT2 system (Thermo Fisher Scientific) or conventional wet transfer. Membranes were incubated with primary Abs at the concentrations specified in Supplemental Table 4. After membrane incubation with an appropriate secondary Ab, the protein of interest was detected by chemical fluorescence following a conventional ECL protocol. Densitometry analysis was performed using the Image Lab software (Bio-Rad).

### Animal studies.

SCLC-N LX33 and SCLC-A LX95 PDX models were provided by Charles M. Rudin and John T. Poirier (MSKCC; refs. 48, 49). Freshly isolated PDX tumors were dissociated into single-cell suspensions using the Human Tumor Dissociation Kit (Miltenyi Biotec) and a gentleMACS Octo Dissociator (Miltenyi Biotec) following the manufacturer’s instructions. After washing cells with PBS 3 times, RBCs were lysed by resuspending cells in 5 mL ACK lysis buffer (Quality Biological). After incubating at room temperature for 5 minutes, the cells were washed with PBS 3 times and resuspended in PBS at 5 ***×*** 10^7^ viable cells per mL. Approximately 5 × 10^6^ viable cells in 100 μL were injected s.c. into the right flanks of 6-week-old NOD-SCID (NOD.Cg-Prkdc scid Il2rg tm1Wjl/SzJ) mice of both genders (Charles River Laboratories).

Once the tumor volume reached 50–100 mm^3^ (average, 14–21 days), mice were randomized into 4 treatment groups to receive the vehicle, NHWD870 or AZD5153, everolimus, or AZD5363, or the combinations via oral gavage. The dosages and frequencies of drug administration are specified in the figure legends. BWs and tumor sizes were measured every 3 days. Treatment was on hold if 1 of the following 2 criteria was met: (a) greater than 15% BW loss compared with the initial BW (at the point of randomization), or (b) active diarrhea. Treatment was resumed once the BW loss recovered to less than 10% reduction of the initial weight and diarrhea had stopped for 3 days. Animals were euthanized if (a) the tumor volume was larger than or equal to 1,500 mm^3^, (b) the tumor became ulcerated, or 3) 75 days had elapsed after a tumor became palpable.

### Histology analysis.

Formalin-fixed tumors were embedded in paraffin wax, and sectioned tissues were stained with H&E or with anticleaved caspase 3 (Cell Signaling Technology, catalog 4850; 1:200) and anti-RSK3 Abs (Novus Biologicals, catalog NBP2-52555; 1:200). Quantification of cleaved caspase 3-positive cells was performed using QuPath (v0.2.2; ref. 50) by counting cytoplasmic staining-positive cells in 4–15 randomly selected images of each tumor.

### RNA isolation from tumor cells of PDX.

Tumor cells were isolated from PDX using a magnetic-activated cell sorting method (51). In brief, after obtaining single-cell suspensions from fresh tumors, cells were centrifuged and resuspended at concentrations of 2 × 10^6^ cells per 80 μL PBS supplemented with 0.5% BSA (Sigma-Aldrich). Afterward, cells were mixed with a 20 μL cocktail from a Mouse Cell Depletion Kit (Miltenyi Biotec). Separation of mouse cells from human tumor cells was achieved using an LS column on a MACS Separator (Miltenyi Biotec). The flow-through fraction, which contained enriched human tumor cells, was collected by centrifugation. The cell pellet was processed for total RNA isolation using an RNeasy Mini Kit (Qiagen).

### RNA-Seq.

mRNA libraries were constructed using a TruSeq Stranded mRNA Library Prep kit V2 (Illumina) and were paired-end sequenced on a HiSeq 4000 system (Illumina) at the NCI Frederick Sequencing core facility.

RNA-Seq reads were processed using the Snakemake-based lcdb-wf transcriptomics pipeline v1.3c (https://github.com/lcdb/lcdb-wf) (52). Briefly, quality was assessed using FastQ Screen and FastQC, and adapter sequences and low-quality bps were trimmed using cutadapt. Trimmed reads were then mapped to the human reference genome (gencode v30) using HISAT2 (v2.1.0), and transcript levels were quantified using the *featureCounts* method from RSubRead (v1.6.4). Differential expression analysis was performed in the R/Bioconductor environment (R v4.0.2; Bioconductor v2.48.0) using the DESeq2 package (v1.28.0) (53, 54). After constructing a DESeqDataSet object from the read counts generated by RSubRead, generalized linear models were fit with default parameters using a treatment coding design (e.g., NHWD870 versus vehicle). Finally, shrunken log-fold changes were estimated for each contrast using the *apeglm* adaptive *t* prior shrinkage approach (55).

### qPCR assay.

After total RNA isolation, RNA was quantified using Nanodrop. Equivalent amounts of total RNA were used for cDNA synthesis using an iScript cDNA Synthesis kit (Bio-Rad). Quantitative analysis of *RPS6KA2* was performed using a SsoAdvanced Universal SYBR Green Supermix (Bio-Rad) and a set of specific primers (*RPS6KA2*, Bio-Rad, qHsaCED0042305; β-actin, Bio-Rad, qHsaCED0036269) on an ABI PRISM7500 real-time PCR system (Thermo Fisher Scientific).

### Statistics.

GraphPad Prism 9.4.1 was used for statistical analysis and graphing. CompuSyn (www.combosyn.com) was used to determine the drug synergy of the low-throughput experiments by calculating CIs (18). Statistical analysis of 2-sample comparisons was performed using the 2-tailed Student’s unpaired *t* test. The 1-way ANOVA with Dunnett’s multiple comparison corrections or the Student’s unpaired *t* test with the FDR multiple comparison corrections was employed for multiple-sample comparisons. The normality and homoscedasticity of the data were examined using the Anderson-Darling test and a SigmaPlot-based test. The log-rank (Mantel-Cox) test was used for Kaplan-Meier survival analyses. A 2-tailed *P* < 0.05 was considered statistically significant. Data are shown as the mean ± SD.

### Study approval.

The animal experiments were approved and performed according to the regulations set by the National Cancer Institute-Bethesda Animal Care and Use Committee.

### Data availability.

All screen results are publicly accessible at https://tripod.nih.gov/matrix-client/ RNA-Seq raw data were deposited in the NCBI’s Gene Expression Omnibus database (GEO GSE155923).

## Author contributions

AK conducted experiments, analyzed data, and revised the manuscript; LG conducted experiments, analyzed data, and revised the manuscript; YJL conducted experiments and analyzed data; VKH analyzed data; XZ, MC, KMW, CKT, LC, CM, and ZI conducted experiments; CJT designed research studies, analyzed data, and revised the manuscript; BAM designed research studies, analyzed data, and revised the manuscript; DSS designed research studies and revised the manuscript; and HC designed and supervised research studies, analyzed data, and wrote and revised the manuscript.

## Supplementary Material

Supplemental data

Supplemental table 1

Supplemental table 2

Supplemental table 3

Supplemental table 4

## Version 1 03/08/2023

Electronic publication
