# Supplementary material for: mTOR inhibition overcomes RSK3-mediated resistance to BET inhibitors in small cell lung cancer
Source: JCI Insight. 2023 Mar 8;8(5):e156657. doi: 10.1172/jci.insight.156657 (PMC10077471; doi:10.1172/jci.insight.156657)
Supplement: Supplemental data [file jciinsight-8-156657-s015.pdf]

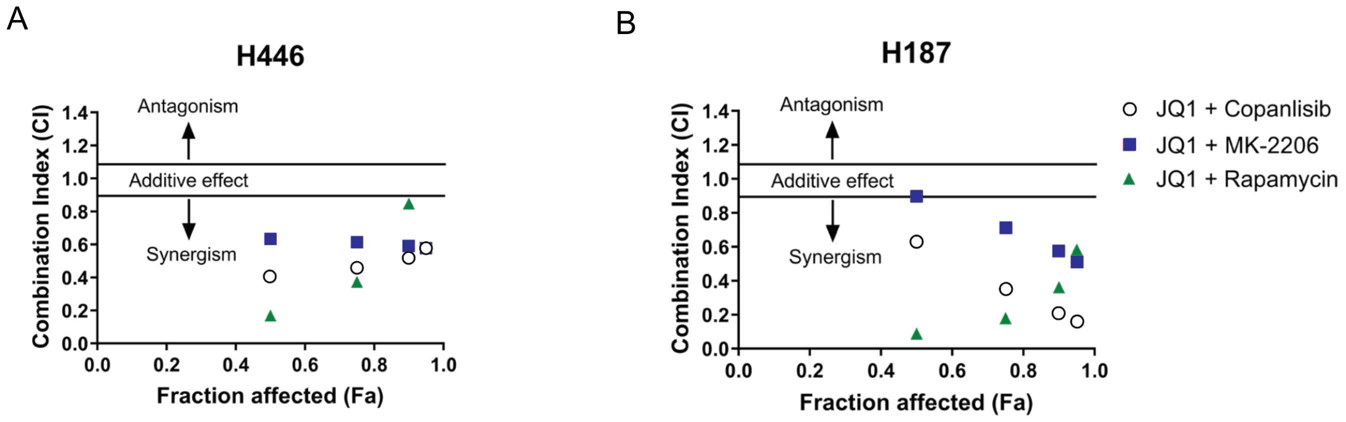

Supplemental Figure S1. The mTORi/BETi combination resulted in a stronger synergy than the AKTi/BETi or the PI-3Ki/BETi combinations. Synergy plots showing CIs versus affected fractions in H446 (SCLC-N subtype; A) and H187 (SCLC-A subtype; B) 72 hours after treatment with JQ1 in combination with copanlisib (PI-3Ki), MK-2206 (AKTi), or rapamycin (mTORi).

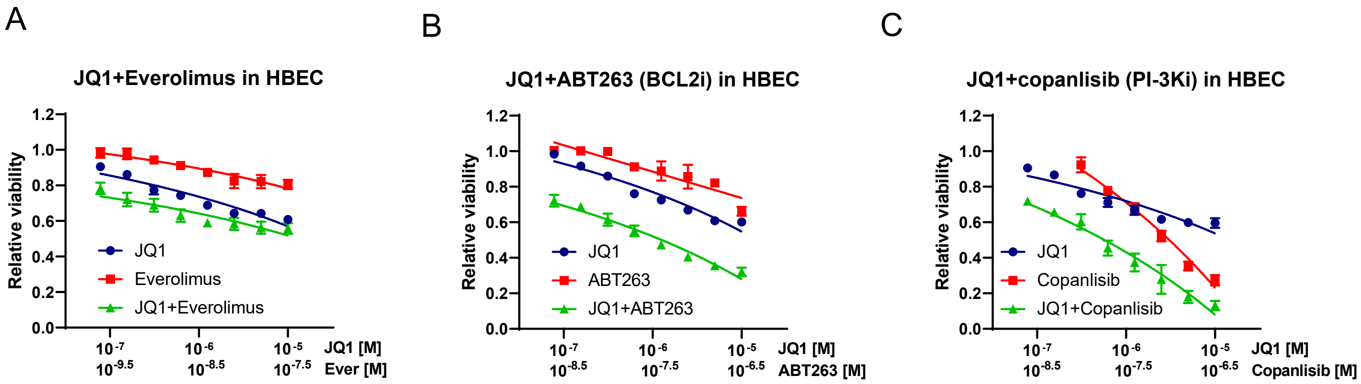

Supplemental Figure S2. Effects of various drug combinations on the viability of immortalized human bronchial epithelial cells (HBEC). The viability of HBEC cells following a 72-hour treatment of JQ1 in combination with everolimus (mTORi; A), ABT263 (BCL2i; B), or copanlisib (PI-3Ki; C) versus single agents.

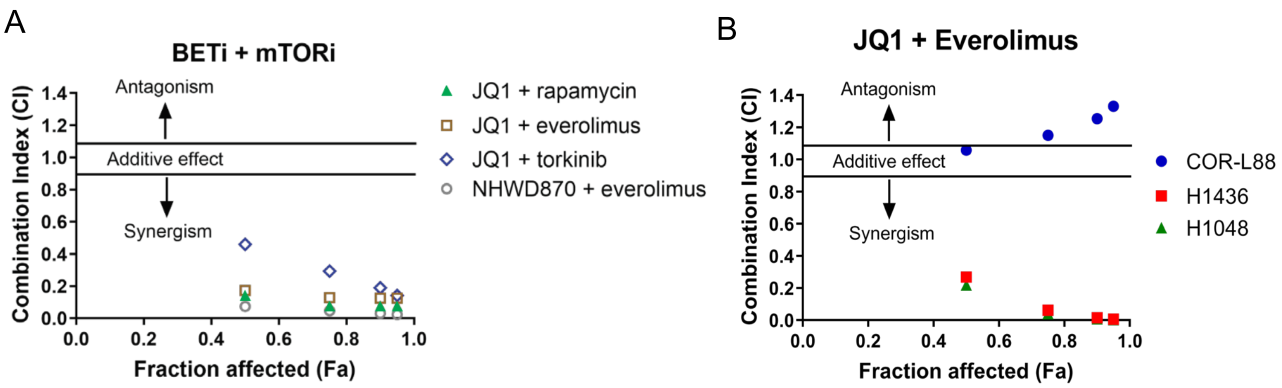

Supplemental Figure S3. The synergy between mTORi and BETi is not restricted to a specific drug or the BETi-sensitive SCLC. (A) Synergy plots showing CIs versus affected fractions in COR-L279 cells following a 72-hour combinatorial treatment of various mTOR and BET inhibitors. **(B)** Synergy plot showing CIs versus affected fractions in three BETi-resistant SCLC lines (SCLC-A subtypes: COR-L88 and H1436; SCLC-Y subtype: H1048) following a 72-hour treatment with the everolimus and JQ1 combination.

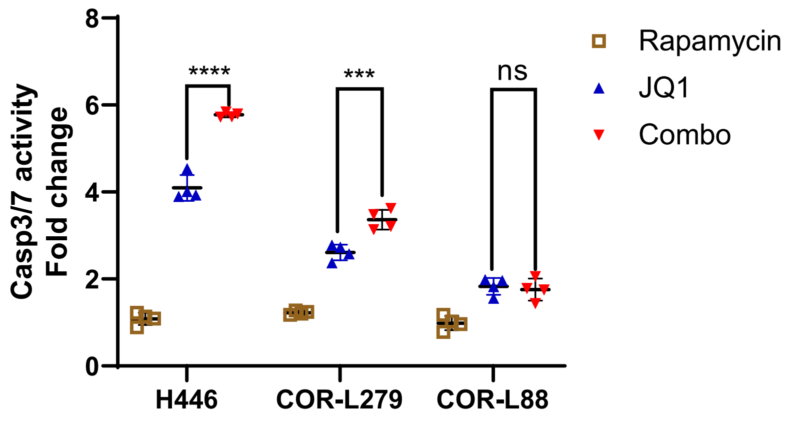

Supplemental Figure S4. Everolimus augments the JQ1-induced apoptosis in H446 and COR-L279 but not in COR-L88 cells. Caspase 3/7 activity was measured at the 48-hour interval in H446, COR-L279, and COR-L88 cells treated with DMSO, rapamycin (50 nmol/L), JQ1 (0.5 micromol/L), and their combination, and the results were presented as the relative changes to untreated cells. The significance of the two-group comparisons was determined using the Student's *t*-test with FDR multiple comparison corrections. ns, not significant; ***, p<0.001; ****, P<0.0001.

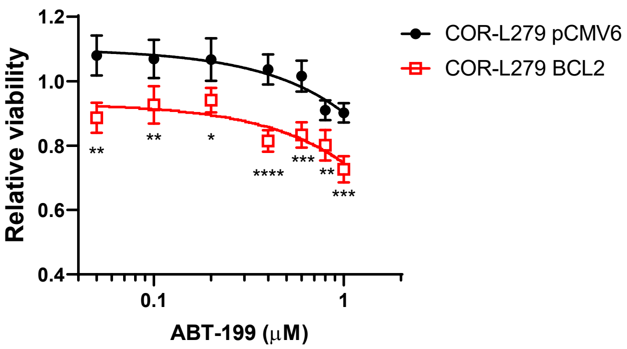

Supplemental Figure S5. Ectopic BCL2 increases the sensitivity of COR-L279 cells to ABT-199, a BCL2 inhibitor. The significance of the two-group comparisons was determined using the Student's *t*-test with the FDR multiple comparison corrections. Error bars represent the SD of four replicates. *, $p<0.05$; **, $p<0.01$; ***, $p<0.001$; ****, $P<0.0001$.

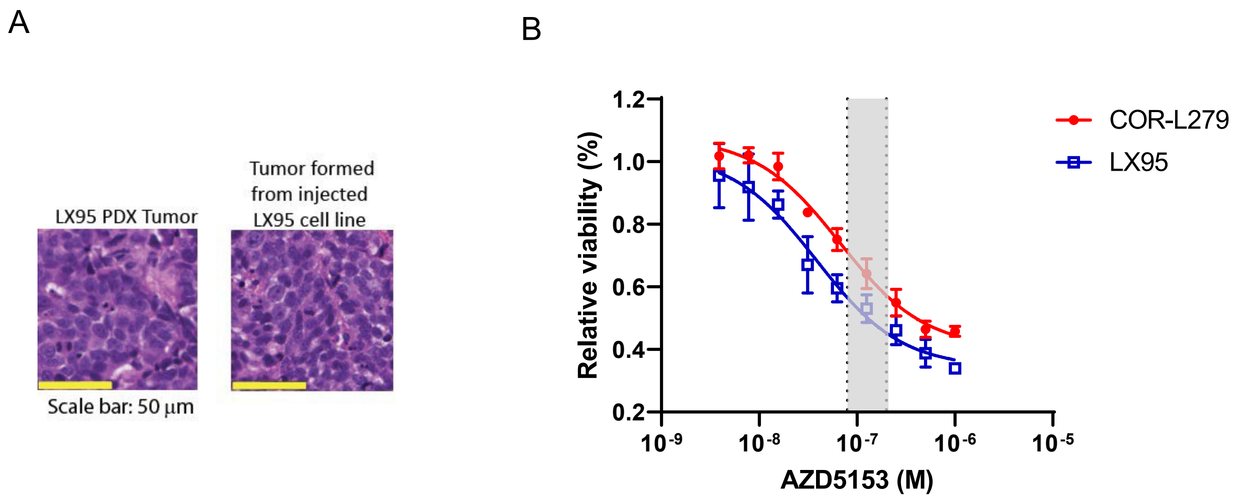

Supplemental Figure S6. The LX95 tumor cell line, established from an SCLC-A subtype PDX, shows exquisite sensitivity to AZD5153 in vitro. (A) A representative histological image of the xenograft tumors formed from the injected LX95 cells (right) was compared to one from LX95 PDX tumor block (left). **(B)** Relative viability of COR-L279 and LX95 cells after a 72-hour treatment of AZD5153. The gray column represents the in vitro dose ranges of AZD5153 (80-200 nmol/L) corresponding to the plasma Cmax in the patients receiving 10-40 mg daily in a phase I trial.

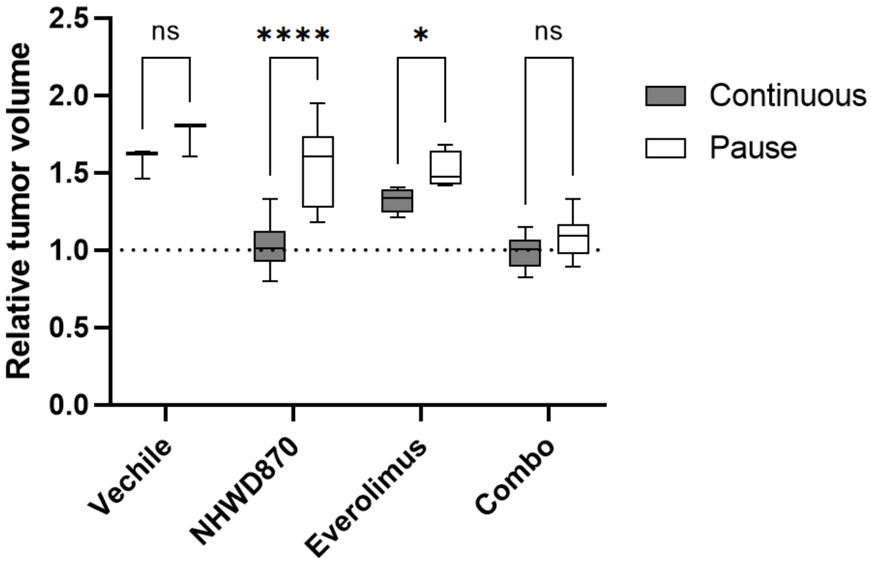

Supplemental Figure S7. A short-term pause of the everolimus/NHWD870 combo treatment did not affect tumor volume control in the LX95 model. Relative tumor volume changes of LX95 tumors in mice receiving continuous treatments of vehicle, everolimus (2 mg/kg daily), NHWD870 (1.5mg/kg, daily), or the combination (NHWD870 1 mg/kg and everolimus 1.5mg/kg, daily) versus those with a 3-day treatment pause. n=3-10 per group. The significance of tumor volume changes between the treatment pause groups and the continuous groups was determined using the Student's *t*-test with the FDR multiple comparison corrections. Whiskers represent minimum to maximum, and boxes show the first, median, and third quartile. The dotted line represents no tumor growth. Where indicated, *, $p<0.05$; ****, $p<0.0001$; ns, not significant.

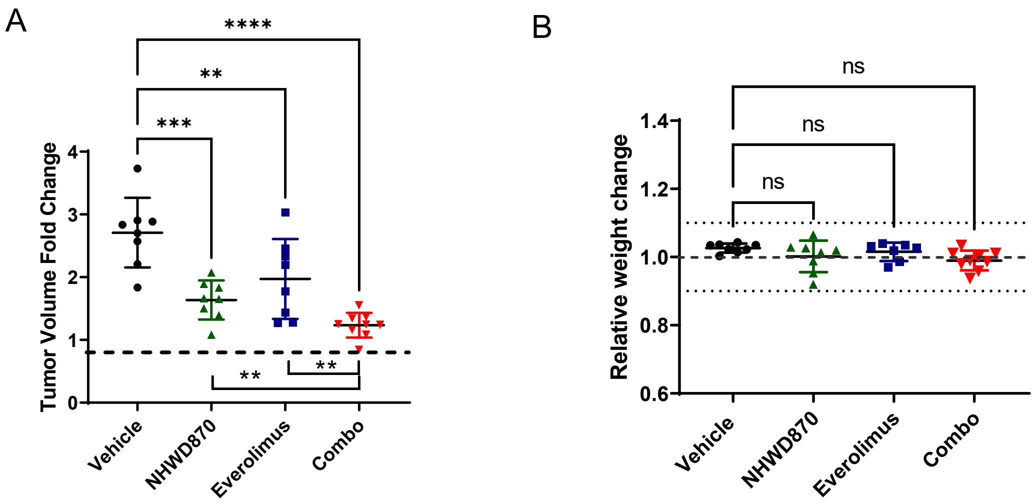

Supplemental Figure S8. Relative tumor volume and body weight changes after one-week treatment of the everolimus/NHWD870 combination in the LX95 PDX model. Relative tumor volume and body weight changes of LX95 PDXs after one-week treatment of vehicle, NHWD870 (1.5mg/kg, daily), everolimus (2mg/kg, daily), or their combination (everolimus 1.5mg/kg and NHWD870 1mg/kg, daily). $n \geq 8$ per group. The significance of the two-group comparisons was determined using the ANOVA test with Dunnett's multiple test correction. Error bars represent SD. ns, not significant; **, $p<0.01$; ***, $p<0.001$; ****, $p<0.0001$.

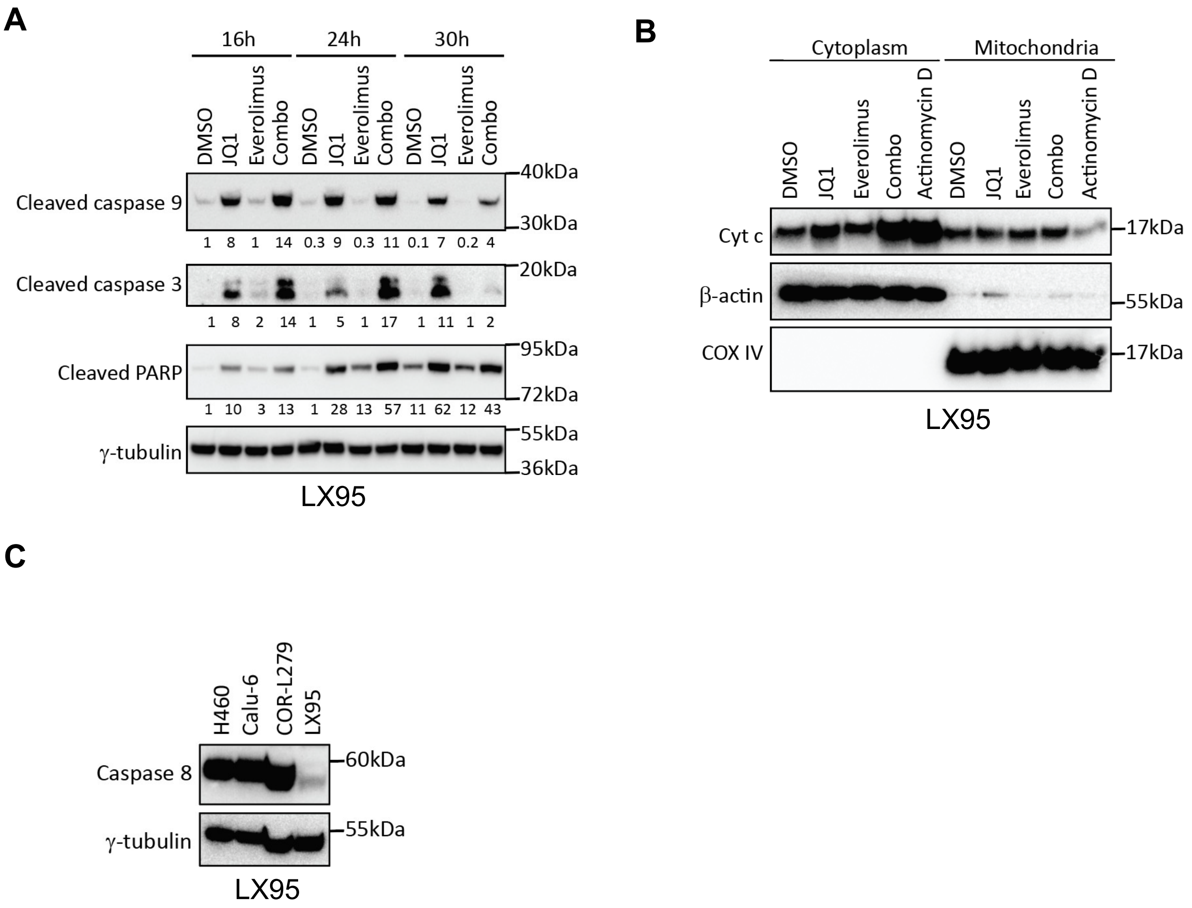

Supplemental Figure S9. Everolimus augments the JQ1-induced apoptosis via the intrinsic apoptotic cascade in LX95 cells. (A) Western blots show cleavage of caspase 9, caspase 3, or PARP at the 16-hour, 24-hour, and 30-hour intervals in LX95 cells treated with JQ1 (156 nmol/L), everolimus (6.25 nmol/L), or the combination. **(B)** Western blots show cytochrome c (Cyt c) abundance in the mitochondrial and cytoplasmic fractions of LX95 cells following a 16-hour treatment of JQ1 (1 micromol/L), everolimus (6.25 nmol/L), or their combination. Actinomycin D (1 microgram/ml, 16 hours) was used as a positive control. **(C)** Western blots showing expression of caspase 8 in two non-small cell lung cancer lines (H460 and Calu-6) and two SCLC lines (SCLC-N: COR-L279 and SCLC-A: LX95).

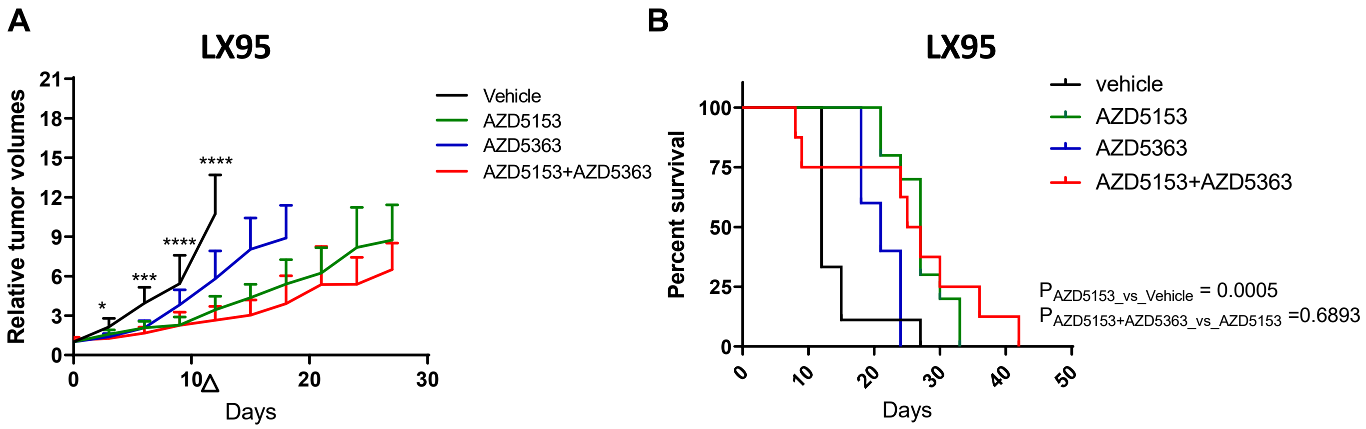

Supplemental Figure S10. The combination of AZD5363 (AKTi) and AZD5153 (BETi) failed to improve tumor control or extend survival in the LX95 PDX model. (A) Relative tumor volume changes within 29 days of drug administration. LX95 PDX mice were initially treated with single-agent AZD5153 (1.5 mg/kg, daily), single-agent AZD5363 (50 mg/kg, daily), or the combination (AZD5153 1 mg/kg and AZD5363 50 mg/kg, daily). Due to premature deaths in the combo group, the dose of AZD5363 in the combination group was reduced to 30mg/kg two weeks after the first dose (triangle). Black asterisks indicate a statistically significant change in tumor volume in the AZD5153 single-agent group compared to Vehicle, as determined by the Student's *t*-test with the FDR multi-comparison corrections. Error bars represent SD. *, $p<0.05$; ***, $p<0.001$; ****, $p<0.0001$. **(B)** Kaplan-Meier survival curves of (A). The significance of the two-group comparisons in (B) was determined by the Log-rank test.

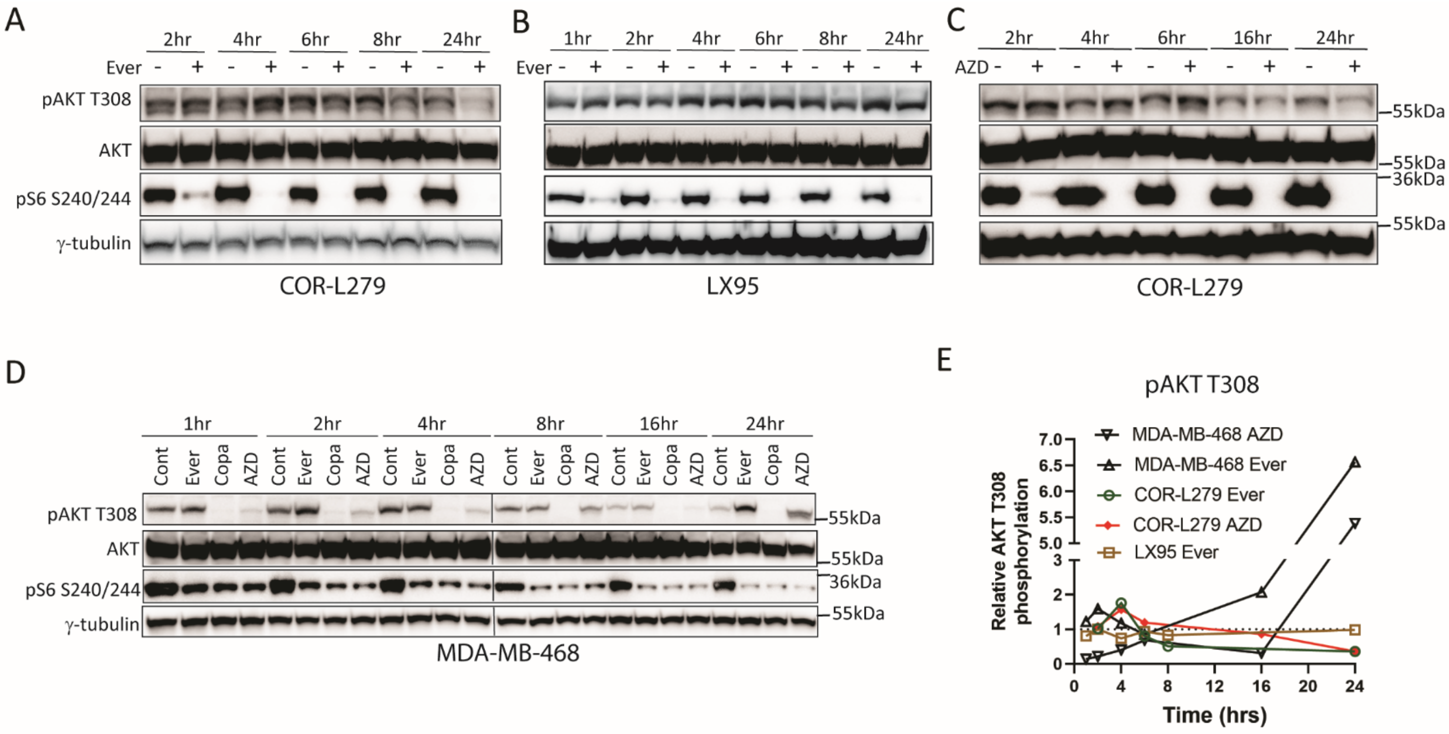

Supplemental Figure S11. mTOR inhibition does not activate the RTK feedback loop in SCLC. (A-C) Western blots show the changes of AKT T308 phosphorylation after treatment of everolimus (5 nmol/L) in COR-L279 (A) and LX95 cells (B), or after treatment of AZD8055 (a mTORi; 500 nmol/L) in COR-L279 cells (C). Phosphorylation of S6 S240/244 was assessed to ensure mTOR inhibition after drug treatment. **(D)** Western blots show the dynamic changes of AKT T308 phosphorylation in MDA-MB-468 cells after treatment with everolimus (5 nmol/L) or AZD8055 (500 nmol/L). Copanlisib (1 micromol/L) was used as a positive control. **(E)** Densitometry of phos-AKT T308 from (A-D). AZD, AZD8055.

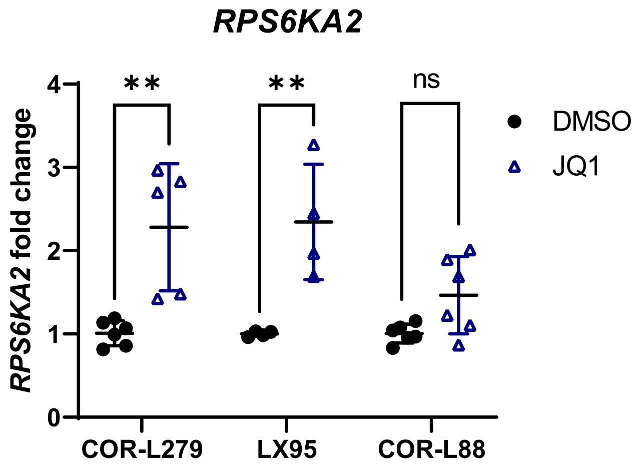

Supplemental Figure S12. JQ1 induces *RPS6KA2* expression in COR-L279 and LX95 but not in COR-L88 cells. COR-L279 and COR-L88 cells were treated with JQ1 (100 nmol/L) for 7 days, while LX95 cells were treated with JQ1 (250 nmol/L) for 24 hours. The significance of the two-group comparisons was determined using the Student's *t*-test with the FDR multiple comparison corrections. Where indicated, **, $p<0.01$; ns, not significant.

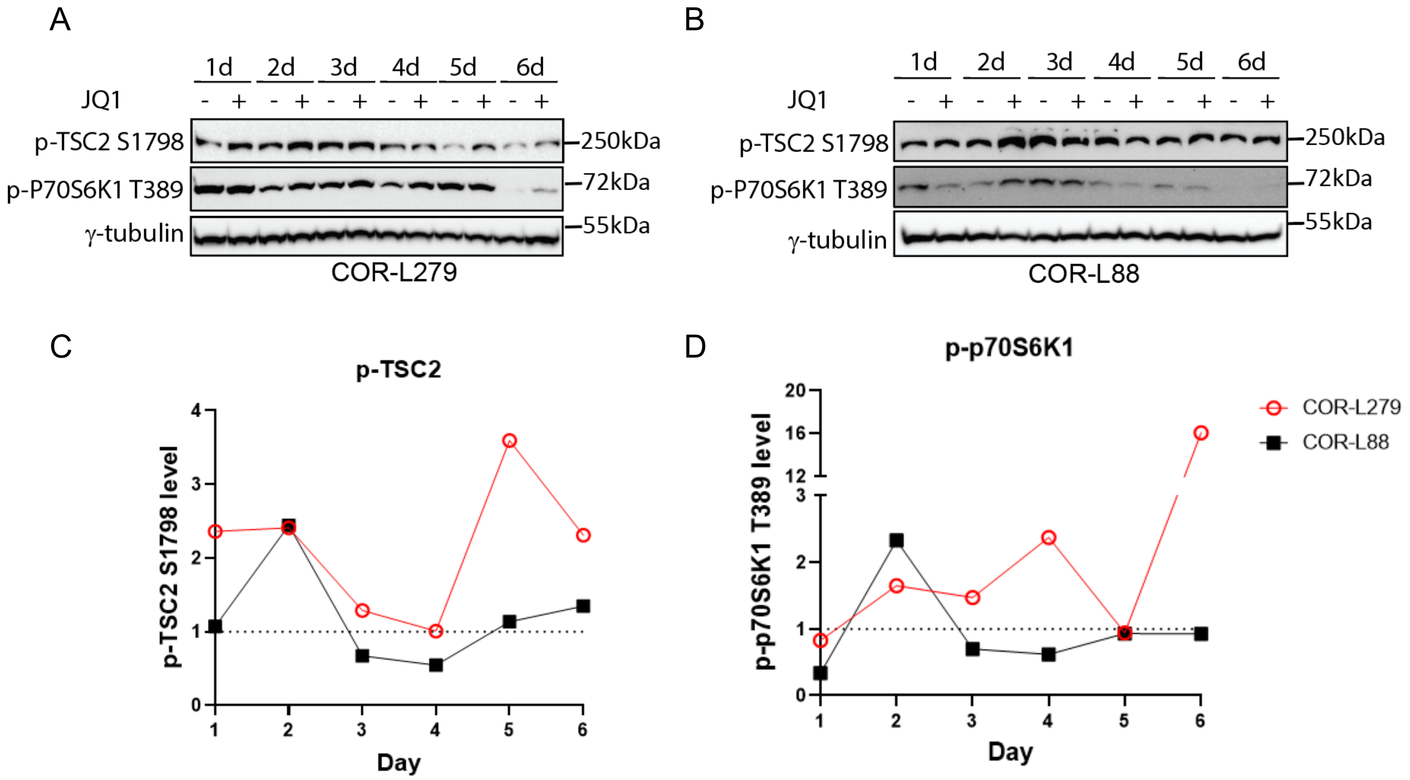

Supplemental Figure S13. The temporal changes of phos-TSC2 S1798 and phos-p70S6K1 T389 in COR-L279 and COR-L88 cells during a 6-day course of JQ1 treatment. COR-L279 (A) and COR-L88 (B) cells were treated with JQ1 (100 nmol/L) or DMSO for six days, and fresh media with the drugs were replenished every 2 days. **(C)** Densitometry of phos-TSC2 S1798 in (A-B). **(D)** Densitometry of phos-p70S6K1 T389 in (A-B).

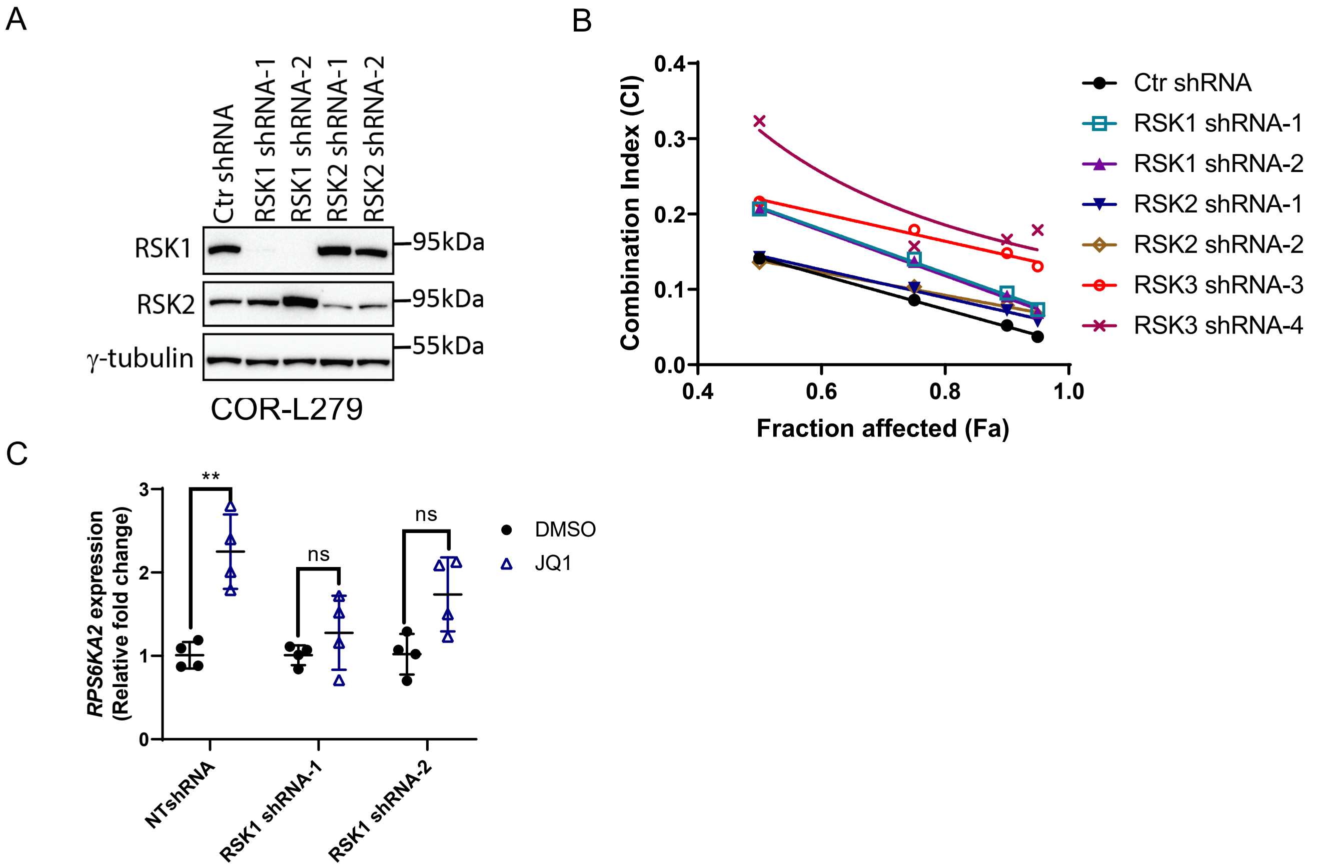

Supplemental Figure S14. RSK1 impacts the synergy of mTORi and BETi in part by regulating the BETi-induced *RPS6KA2* expression. (A) Western blots showing knockdown of RSK1 and RSK2 in COR-L279 cells by shRNAs. **(B)** A synergy plot comparing the combination index of the JQ1 and rapamycin combo in the COR-L279 stably expressing shRNA targeting RSK1, RSK2, or RSK3. **(C)** Effect of RSK1 knockdown on the *RPS6KA2* induction by JQ1 (0.5 micromol/L, 24 hours) in COR-L279 cells. The significance of the two-group comparisons was determined using the Student's *t*-test with the FDR multiple comparison correction. **, p<0.01; ns, not significant.

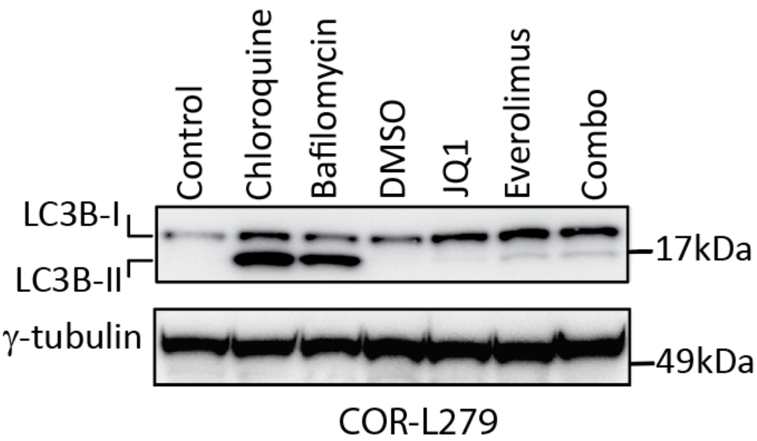

Figure S15. JQ1 does not affect the everolimus-induced autophagy in COR-L279 cells. COR-L279 cells were treated with JQ1 (1 micromol/L), everolimus (6.25 nmol/L), or the combination for 24 hours before measurement of the LC3B-I and LC3B-II levels. Chloroquine (200 micromol/L, 24 hours) and bafilomycin (150 nmol/L, 24 hours) were used as positive controls.

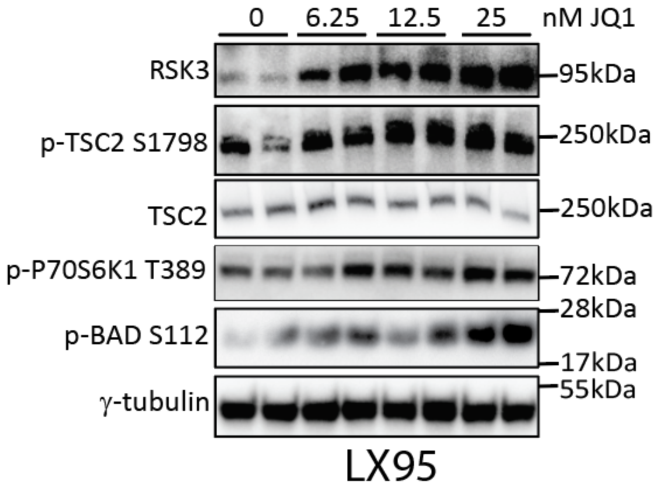

Supplemental Figure S16. JQ1 induces phos-BAD S112 by activating the RSK3-TSC2-p70S6K1 cascade in LX95 cells. Western blots show the alterations in RSK3, phos-TSC2 S1798, phos-P70S6K1 T389, and phos-BAD S112 expression in LX95 cells following one-week treatment of JQ1 at the specified doses. Fresh media and JQ1 were replenished every three days. Two experimental replicates were used.
