# Supplementary material for: mTOR inhibition overcomes RSK3-mediated resistance to BET inhibitors in small cell lung cancer
Source: JCI Insight. 2023 Mar 8;8(5):e156657. doi: 10.1172/jci.insight.156657 (PMC10077471; doi:10.1172/jci.insight.156657)
Supplement: Supplemental table 2 [file jciinsight-8-156657-s017.pdf]

Supplemental Table S2. The targets and the concentration ranges of the drugs showing synergy with JQ1 in ≥ 3 SCLC cell lines in the 10x10 screens.

Name	Target pathway(s)	Dose range tested
Rapamycin (sirolimus)	mTOR (mTORC1)	39.06-40,000nmol/L
Everolimus	mTOR (mTORC1)	39.06-40,000nmol/L
Ridaforolimus	mTOR (mTORC1)	39.06-40,000nmol/L
GDC-0980 (Apitolisib)	Both PI-3K and mTORC1/2	39.06-40,000nmol/L
Copanlisib	PI-3K	15.63-4,000nmol/L
ZSTK-474	PI-3K	39.06-40,000nmol/L
MK-2206	AKT	39.06-40,000nmol/L
PHA-793887	CDK2/1/4	39.06-40,000nmol/L
Milciclib	CDK2 and tropomyosin receptor kinase (TRK) A	39.06-40,000nmol/L
Dinaciclib (SCH727965)	CDK1/2/5/9	1.95-500nmol/L
Navitoclax (ABT-263)	Bcl-2 and Bcl-xL	39.06-40,000nmol/L
Doxorubicin	DNA	19.53-5,000nmol/L
Afatinib	Epidermal growth factor receptor (EGFR)	39.06-40,000nmol/L
MLN-4924	NEDD8-Activating Enzyme	39.06-40,000nmol/L
Carfilzomib	Proteasome	3.91-1,000nmol/L
Tanespimycin	HSP90	39.06-40,000nmol/L
Danusertib	Pan-Aurora kinase and ABL kinase	19.53-5,000nmol/L
ARRY-520	Kinesin spindle protein	0.78-200nmol/L
OTS-167	Maternal embryonic leucine zipper kinase (MELK)	3.91-1,000nmol/L
Tamoxifen	Estrogen receptor	39.06-40,000nmol/L
Mocetinostat	histone deacetylase 1 (HDAC1)	39.06-40,000nmol/L
Belinostat	histone deacetylase (HDAC)	39.06-40,000nmol/L
