# Supplementary material for: mTOR inhibition overcomes RSK3-mediated resistance to BET inhibitors in small cell lung cancer
Source: JCI Insight. 2023 Mar 8;8(5):e156657. doi: 10.1172/jci.insight.156657 (PMC10077471; doi:10.1172/jci.insight.156657)
Supplement: Supplemental table 4 [file jciinsight-8-156657-s019.pdf]

Supplemental Table S4. The list of the antibodies used in this study.

Antibody target	Vendor and catalog number	Dilution
β-actin	Santa Cruz Biotechnology, #sc-47778	1:2,000
AKT	Cell Signaling Technology, #2920	1:1,000
Phos-AKT T308	Cell Signaling Technology, #4056	1:1,000
BAD	Cell Signaling Technology, #9239	1:1,000
Phos-BAD S112	Cell Signaling Technology, #5284	1:1,000
Phos-BAD S136	Cell Signaling Technology, #4366	1:1,000
Phos-BAD S155	Cell Signaling Technology, #9297	1:1,000
BID	Cell Signaling Technology, #2002	1:1,000
BCL2	Santa Cruz Biotechnology, #SC-7382	1:200
Cleaved caspase 3	Cell Signaling Technology, #9661	1:1,000
Caspase 3	Cell Signaling Technology, #9662	1:1,000
Cleaved caspase 8	Cell Signaling Technology, #9496	1:1,000
Caspase 8	Cell Signaling Technology, #4790	1:1,000
Cleaved caspase 9	Cell Signaling Technology, #7237	1:1,000
Caspase 9	Cell Signaling Technology, #9502	1:1,000
Cleaved caspase 3	Cell Signaling Technology, #9661	1:1,000
Caspase 3	Cell Signaling Technology, #9662	1:1,000
Cleaved PARP	Cell Signaling Technology, #9542	1:1,000
PARP	Cell Signaling Technology, #9662	1:1,000
COX IV	Cell Signaling Technology, #4850	1:1,000
Cytochrome C	Abcam, #Ab110325	1:2,000
HA-tag	Cell Signaling Technology, #3724	1:1,000
LC-3B	Cell Signaling Technology, #3868	1:1,000
NaK-ATPase	Santa Cruz Biotechnology, #sc-71638	1:1,000
p70S6K1	Cell Signaling Technology, #9202	1:1,000
Phos-p70S6K1 T389	Cell Signaling Technology, #9205	1:1,000
RSK3	Novus Biologicals, #NBP2-52555	1:1,000
RSK3	Origene, # TA809889	1:1,000
Phos-TSC Ser1798	Santa Cruz Biotechnology, #sc-293149	1:1,000
phos-S6 S240/244	Cell Signaling Technology, #5364	1:1,000
TSC2	Cell Signaling Technology, #4308	1:1,000
γ-tubulin	Sigma, #T6557	1:10,000
γ-tubulin	Abcam, #Ab11316	1:5,000
